# FAM3A mediates PPARγ's protection in liver ischemia-reperfusion injury by activating Akt survival pathway and repressing inflammation and oxidative stress

**DOI:** 10.18632/oncotarget.17805

**Published:** 2017-05-11

**Authors:** Zhenzhen Chen, Junpei Wang, Weili Yang, Ji Chen, Yuhong Meng, Bin Geng, Qinghua Cui, Jichun Yang

**Affiliations:** ^1^ Department of Physiology and Pathophysiology, School of Basic Medical Sciences Key Laboratory of Molecular Cardiovascular Sciences of the Ministry of Education Center for Non-coding RNA Medicine, Peking University Health Science Center, Beijing 100191, China; ^2^ Department of Biomedical Informatics, School of Basic Medical Sciences Key Laboratory of Molecular Cardiovascular Sciences of the Ministry of Education, Center for Non-coding RNA Medicine, Peking University Health Science Center, Beijing 100191, China; ^3^ State Key Laboratory of Cardiovascular Disease, Fuwai Hospital of Chinese Academy of Medical Sciences, Peking Union Medical College, Beijing 100037, China

**Keywords:** liver ischemia/reperfusion injury, FAM3A, PPARgamma, Akt, FOXO1

## Abstract

FAM3A is a novel mitochondrial protein, and its biological function remains largely unknown. This study determined the role and mechanism of FAM3A in liver ischemia-reperfusion injury (IRI). In mouse liver after IRI, FAM3A expression was increased. FAM3A-deficient mice exhibited exaggerated liver damage with increased serum levels of AST, ALT, MPO, MDA and oxidative stress when compared with WT mice after liver IRI. FAM3A-deficient mouse livers had a decrease in ATP content, Akt activity and anti-apoptotic protein expression with an increase in apoptotic protein expression, inflammation and oxidative stress when compared WT mouse livers after IRI. Rosiglitazone pretreatment protected against liver IRI in wild type mice but not in FAM3A-deficient mice. In cultured hepatocytes, FAM3A overexpression protected against, whereas FAM3A deficiency exaggerated oxidative stress-induced cell death. FAM3A upregulation or FAM3A overexpression inhibited hypoxia/reoxygenation-induced activation of apoptotic gene and hepatocyte death in P2 receptor-dependent manner. FAM3A deficiency blunted rosiglitazone's beneficial effects on Akt activation and cell survival in cultured hepatocytes. Collectively, FAM3A protects against liver IRI by activating Akt survival pathways, repressing inflammation and attenuating oxidative stress. Moreover, the protective effects of PPARγ agonist(s) on liver IRI are dependent on FAM3A-ATP-Akt pathway.

## INTRODUCTION

Liver ischemia reperfusion injury (IRI) happens when ischemia is followed by the blood supply immediately after liver surgery. Liver IRI promotes the death of hepatocytes and leads to liver structural damage [1, 2]. Liver IRI is a major complication of liver tumor resection, transplantation, hypovolemic shock and other liver surgeries [3]. Several mechanisms including adenosine triphosphate (ATP) depletion, reactive oxygen species (ROS) overproduction, macrophage activation and inflammation had been proposed to explain the pathogenesis of liver IRI [4, 5]. Recently, it had been realized that peroxisome proliferator activated receptor gamma (PPARγ) plays important roles in liver IRI [6–8]. In the liver with IRI, PPARγ expression and activity are increased, which protects against liver damage [6]. Deficiency of PPARγ aggravates, whereas PPARγ activation induced by rosiglitazone, pioglitazone, epidermal growth factor(EGF) and insulin like growth factor-1(IGF-1) ameliorates liver IRI in animal models [9–11]. However, the protective mechanism(s) of PPARγ activation in liver IRI still remains unclear [11].

FAM3A is one member of family with sequence similarity 3 (FAM3) gene family [12]. More recently, we had revealed that FAM3A is a direct target gene of PPARγ [13]. FAM3A protein is located in mitochondria, and enhances ATP production and release to promote Akt activation in various cell types [14, 15]. Given the important roles of Akt activity [16, 17] in the protection of liver IRI, it is reasonable to speculate that FAM3A may be involved in liver IRI process. More importantly, as a direct target gene of PPARγ, whether or not FAM3A is involved in PPARγ's protection in liver IRI is a critical issue for understanding the protective mechanism(s) of the PPARγ agonists against liver IRI.

In the current study, we demonstrated that knockdown or deficiency of hepatic FAM3A markedly exaggerated liver IRI. FAM3A protects against liver IRI via activation of Akt survival pathways, attenuation of inflammation and oxidative stress. Moreover, the beneficial effects of PPARγ activation on liver IRI is dependent on FAM3A and its downstream signaling pathways.

## RESULTS

### FAM3A expression was increased in mouse liver after IRI

To determine whether FAM3A is involved in the pathogenesis of liver IRI, a mouse model of 70% liver IRI was generated. The mRNA level of FAM3A was increased in IRI liver (Supplementary Figure 1A). Western blotting and immunohistochemical staining assays revealed that both PPARγ and FAM3A protein expression were increased in IRI liver (Supplementary Figure 1B-1C). In IRI mouse liver, pFOXO1 level was decreased with an increase in FOXO1 level (Supplementary Figure 1B). Caspase 3 was also activated in IRI mouse lives (Supplementary Figure 1B). Moreover, nuclear factor kappa B (NFκB) expression was increased with a decrease in cytosolic IκBα (Supplementary Figure 1D). In both sham and IRI mouse livers, FAM3A protein is mainly located in mitochondrial fraction (Supplementary Figure 2). In contrast, western blotting and immunohistochemical staining assays indicated that FAM3B, FAM3C and FAM3D remained unchanged in IRI mouse livers (Supplementary Figure 3A-3B), suggesting that they may not be involved in liver IRI.

### Knockdown of hepatic FAM3A exaggerated liver IRI

**Figure 1:**
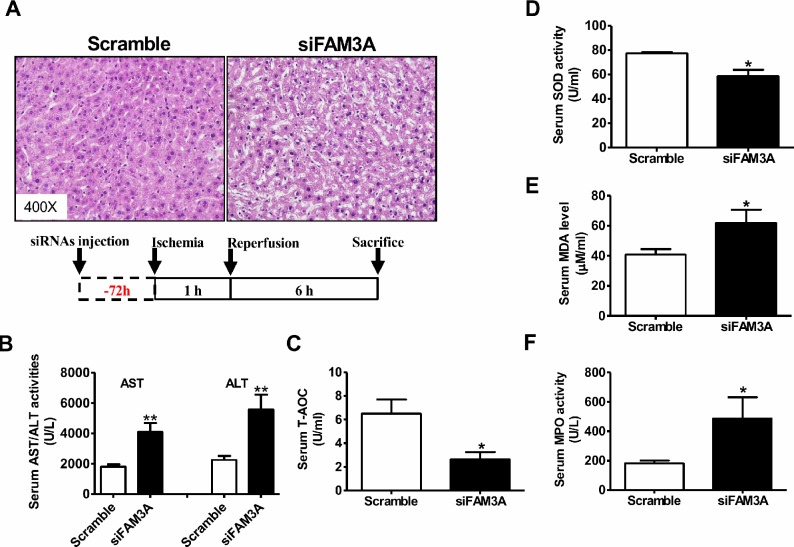
Knockdown of FAM3A exaggerated liver IRI in mouse The mice were treated with siFAM3A or scrambled siRNA via tail-vein injection of siRNA. 72 hours post injection, the mice were subjected to liver IRI. **(A)** Representative images of H.E stained livers. The magnifying power had been marked in the images. **(B)** Silencing of FAM3A elevated serum AST and ALT activities. N=18, **p<0.01 versus control mice treated with scrambled siRNA. **(C-D)** Silencing of hepatic FAM3A reduced T-AOC **(C)** and SOD activity **(D)** in serum of IRI mice. **(E-F)** Silencing of hepatic FAM3A elevated MDA level **(E)** and MPO **(F)** activity in serum of IRI mice. IRI, ischemia/reperfusion injury; AST, aspartate aminotransferase; ALT, alanine aminotranferase; T-AOC, total anti-oxidative capacity; SOD, superoxide dismutase; MDA, methane dicarboxylic aldehyde; MPO, myeloperoxidase. N=8-10, *p<0.05 versus control mice.

**Figure 2:**
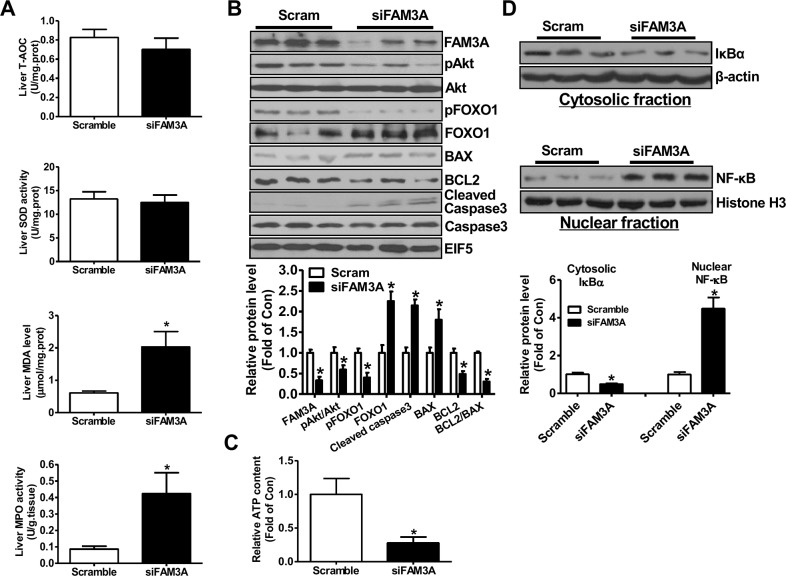
Knockdown of FAM3A increased oxidative stress and activated apoptotic gene in IRI mouse livers **(A)** Silencing of FAM3A on T-AOC, SOD activity, MDA level and MPO activity in IRI mouse livers. **(B)** Silencing of FAM3A on apoptotic protein levels in IRI mouse livers. Representative gel images were shown in upper panel, and quantitative data in lower panel. **(C)** Silencing of FAM3A reduced ATP content in IRI mouse livers. **(D)** Silencing of FAM3A on cytosolic expression of IκBα and nuclear expression of NF-κB in IRI mouse livers. Representative gel images were shown in upper panel, and quantitative data shown in lower panel. N=6-8, *p<0.05 versus control mice.

To directly determine the role of FAM3A in liver IRI, hepatic FAM3A was first knockdown by tail-vein injection of siFAM3A (scrambled siRNA as control), and then the mice were subjected to IRI at 72 hours post siRNA treatment. H.E staining revealed that siFAM3A-treated mice exhibited more severe liver damage than control mice treated with scrambled siRNA (Figure 1A). siFAM3A-treated mice displayed a significant increase in serum AST and ALT activities than control mice (Figure 1B). Myeloperoxidase (MPO) is a functional marker of neutrophil activation, whereas malondialdehyde (MDA) is a product of lipid peroxidation induced by free radicals [18]. MDA will damage mitochondrial membrane and promotes cell death, and is a biomarker of oxidative stress [19]. MPO and MDA are also well recognized as biomarkers for evaluation of liver IRI beyond AST and ALT activities. siFAM3A treatment reduced serum total anti-oxidative capacity (T-AOC) and superoxide dismutase (SOD) activity (Figure 1C–1D), and increased serum MDA level and MPO activity (Figure 1E–1F). In IRI mouse liver, siFAM3A treatment significantly elevated MDA level and MPO activity without significant impact on T-AOC and SOD activity (Figure 2A). siFAM3A treatment significantly reduced the mRNA and protein (Figure 2B, Supplementary Figure 4A-4B) levels of FAM3A, and ATP content (Figure 2C) in IRI mouse liver. With the decrease in FAM3A expression and ATP content, pAkt and pFOXO1 protein levels were decreased, whereas FOXO1 protein level was increased in IRI liver. In IRI mouse liver with FAM3A silencing, anti-apoptotic protein BCL-2 was decreased, whereas apoptotic BAX was increased, resulting in a marked decrease in BCL-2/BAX ratio (Figure 2B). Consistently, the mRNA levels of BAX, BIM and FOXO1 were increased, whereas those of BCL-2 and murine double mimute2 (MDM2) were reduced in liver after FAM3A inhibition (Supplementary Figure 4C). FAM3A inhibition also increased active caspase 3 level in IRI mouse liver (Figure 2B). Moreover, FAM3A knockdown decreased cytosolic protein level of IκBα with a significant increase in nuclear NF-κB expression (Figure 2D). In support, the mRNA levels of tumor necrosis factor alpha (TNF-α), interleukin 1 alpha (IL-1α), monocyte chemotactic protein 1 (MCP-1) and interferon gamma-induced protein 10 (IP-10) were increased in IRI liver after FAM3A inhibition (Supplementary Figure 4D). Overall, these findings revealed that silencing of FAM3A exaggerated liver IRI.

### Activation of FAM3A-ATP-Akt pathway is involved in rosiglitazone's beneficial effects on liver I/R injury

To evaluate whether upregulation of FAM3A expression is associated with attenuated liver IRI by rosiglitazone, mice were orally administered with rosiglitazone for 24 hours, and then subjected to liver IRI. H.E staining revealed that rosiglitazone pretreatment significantly attenuated IRI with reduced serum AST and ALT activities (Supplementary Figure 5A-5B). Rosiglitazone pretreatment upregulated FAM3A mRNA and protein levels, and increased ATP content in mouse liver (Supplementary Figure 5C-5F, Supplementary Figure 6). With FAM3A upregulation, pAkt and pFOXO1 levels were increased with a decrease in FOXO1 protein level in IRI mouse liver (Supplementary Figure 5C-5D). Rosiglitazone reduced the mRNA level of inflammatory cytokines and increased mRNA level of BCL-2 in IRI mouse liver (Supplementary Figure 5E). Overall, PPARγ's protection in liver IRI is associated with the upregulation of FAM3A.

### FAM3A deficiency exaggerated liver IRI

To further confirm the protective effects of FAM3A in liver IRI, FAM3A^−/−^ mice were generated using TALEN technology. One nucleotide was deleted in exon 4 of mouse FAM3A gene, resulting in early termination of FAM3A mRNA translation (Supplementary Figure 7A). Genomic DNA (Supplementary Figure 7B) and hepatic FAM3A mRNA (Supplementary Figure 7C) sequencing validated the successful generation of FAM3A^−/−^ mice. Western blotting assays confirmed the deficiency of FAM3A protein in FAM3A^−/−^ mouse livers (Supplementary Figure 7D). At physiological condition, FAM3A^−/−^ mice exhibit normal liver structure (Supplementary Figure 8A), serum AST and ALT activities (Supplementary Figure 8B) and liver weight (data not shown) as wild type (WT) mice. Although FAM3A^−/−^ mouse livers exhibit lower ATP content, they have comparable mRNA levels of inflammatory and apoptotic genes (Supplementary Figure 8C-8E) as WT mouse livers. Moreover, FAM3A^−/−^ mice displays similar T-AOC, SOD and MPO activities, and MDA level in serum and liver as WT mice in physiological condition (data not shown).

**Figure 3:**
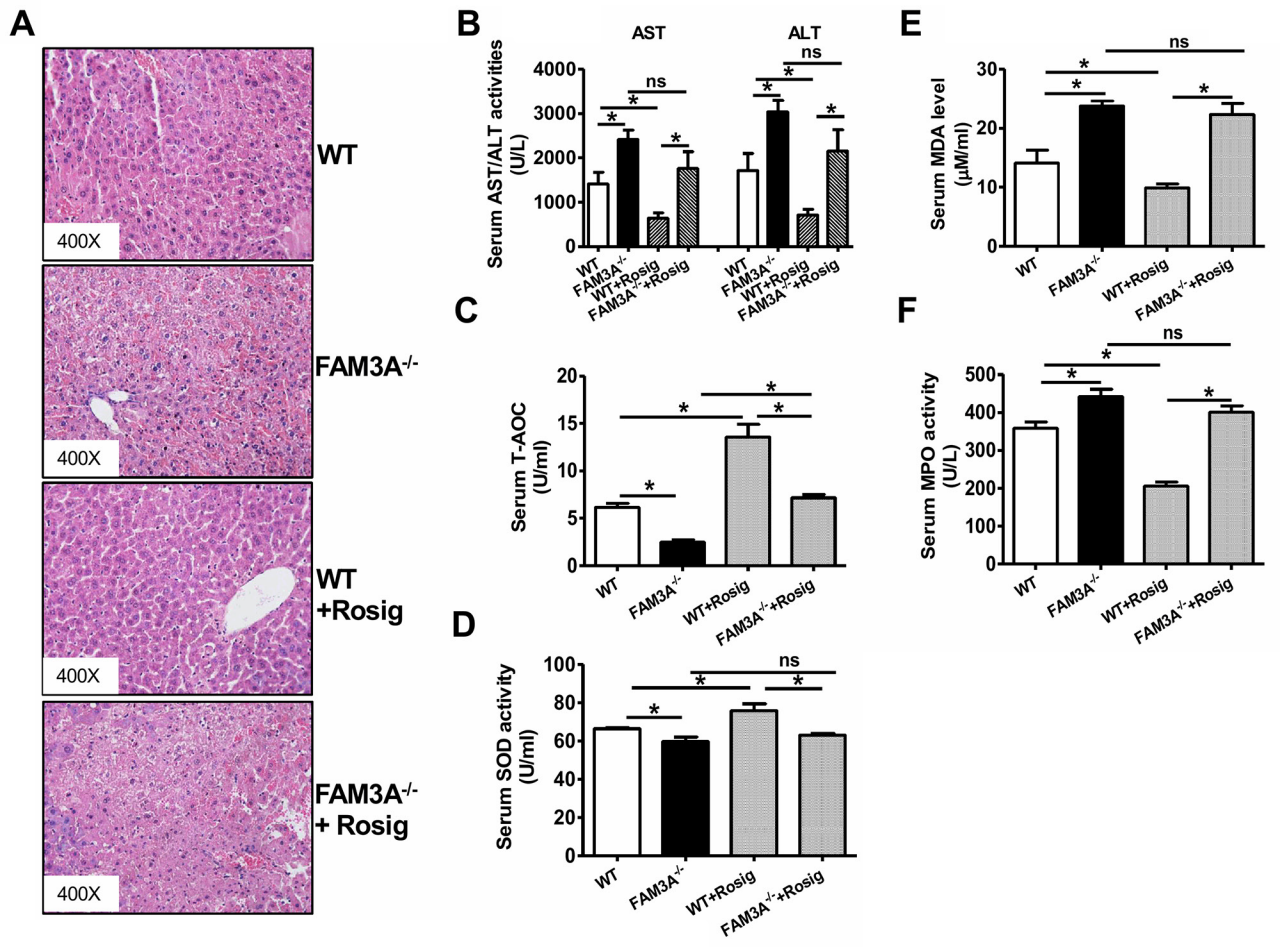
Deficiency of FAM3A abolished PPARγ's protective effects in liver IRI WT or FAM3A^−/−^ mice were orally pretreated with rosiglitazone for 36 hours, and then subjected to liver IRI. **(A)** Representative images of H.E stained livers. **(B)** Deficiency of FAM3A elevated serum AST and ALT activities. **(C-D)** Deficiency of FAM3A reduced T-AOC **(C)** and SOD activity **(D)** in serum of IRI mice with or without rosiglitazone treatment. E-F) Deficiency of FAM3A elevated MDA level **(E)** and MPO **(F)** activity in serum of IRI mice. Rosiglitazone protected against liver IRI in WT mouse, but not in FAM3A^−/−^ mice. WT, wild type mice; FAM3A^−/−^, FAM3A-deficient mice; WT+Rosig, wild type mice pretreated with rosiglitazone; FAM3A^−/−^+Rosig, FAM3A-deficient mice pretreated with rosiglitazone. N=8-10, *p<0.05 between indicated two groups; ns, *no statistically* significant *difference* between indicated groups.

**Figure 4:**
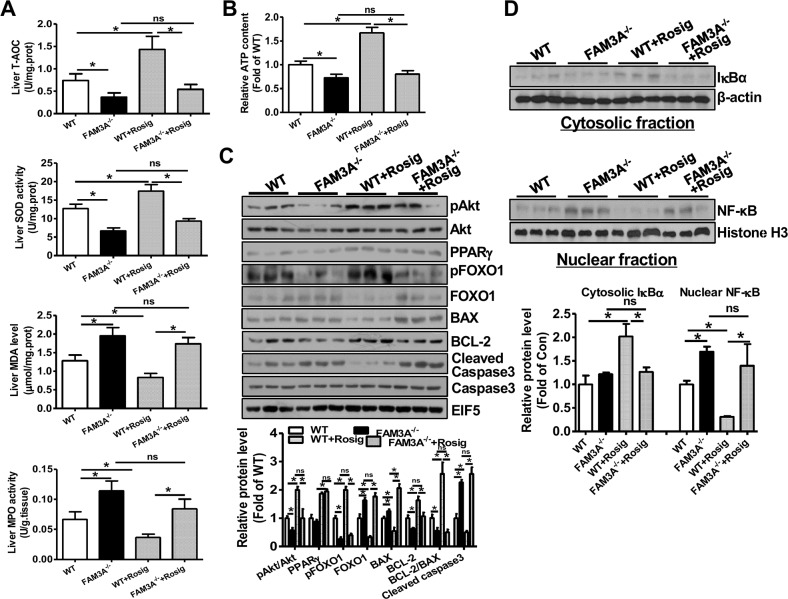
PPARγ activation failed to activate Akt pathway, and repress inflammation and oxidative stress in FAM3A^−/−^ mouse livers with IRI **(A)** Deficiency of FAM3A on T-TOC, SOD activity, MDA level and MPO activity in mouse livers with or without rosiglitazone pretreatment. **(B)** Rosiglitazone pretreatment failed to elevate cellular ATP content in FAM3A^−/−^ mouse livers with IRI. **(C)** Deficiency of FAM3A abolished the beneficial effects of PPARγ activation on apoptotic protein levels. Representative gel images were shown in upper panel, and quantitative data shown in lower panel. **(D)** PPARγ activation failed to repress nuclear NF-κB activity in FAM3A^−/−^ mouse livers with IRI. Representative gel images were shown in upper panel, and quantitative data shown in lower panel. N=8-10, *p<0.05 between indicated two groups; ns, *no statistically* significant *difference* between indicated groups.

WT and FAM3A^−/−^ mice were pretreated with or without rosiglitazone for 24 hours, and then subjected to liver IRI. When compared to WT mice, FAM3A^−/−^ mice displayed exaggerated liver injury (Figure 3A), and had higher serum AST and ALT activities (Figure 3B). FAM3A^−/−^ mice displayed decreased T-AOC and SOD activity, and increased MDA level and MPO activity in serum after liver IRI (Figure 3C–3F). Rosiglitazone pretreatment significantly reduced serum activities of AST/ALT and MPO, MDA level, and increased serum T-AOC and SOD activities in WT mice (Figure 3B–3F). However, the protective effect of rosiglitazone on liver IRI was abolished in FAM3A^−/−^ mice (Figure 3A–3F). In the liver, FAM3A^−/−^ mice displayed a significant reduction in T-AOC and SOD activity with an increase in MDA level and MPO activity when compared with WT mice (Figure 4A). Rosiglitazone pretreatment increased T-AOC and SOD activity, and reduced MDA level and MPO activity in WT mouse livers, but not in FAM3A^−/−^ mouse livers with IRI (Figure 4A). Moreover, glutamate dehydrogenase (GDH) activity and nitric oxide (NO) level was increased in IRI mouse livers (Supplementary Figure 9A-9B), suggesting that mitochondrial function was impaired after IRI. FAM3A^−/−^ mouse livers exhibited higher GDH activity and NO level than WT mouse livers after IRI (Supplementary Figure 9C-9D). Rosiglitazone pretreatment reduced GDH activity and NO level in WT but not in FAM3A^−/−^ mouse livers (Supplementary Figure 9C-9D). FAM3A^−/−^ mouse livers exhibited reduced cellular ATP content, and rosiglitazone failed to elevate its level (Figure 4B). When compared to WT mouse livers, FAM3A^−/−^ mouse livers had reduced pAkt, pFOXO1 and BCL-2 protein levels with increased FOXO1, BAX and active caspase 3 protein levels after IRI (Figure 4C). Rosiglitazone pretreatment repressed these abnormalities in WT mouse livers, but not in FAM3A^−/−^ mouse livers (Figure 4C). FAM3A^−/−^ mouse livers had similar PPARγ mRNA (Supplementary Figure 10A) and protein levels (Figure 4C) as WT mouse livers. Rosiglitazone administration similarly elevated the mRNA and protein levels of PPARγ in WT and FAM3A^−/−^ mouse livers (Supplementary Figure 10A, Figure 4C). FAM3A^−/−^ mouse livers had increased mRNA levels of BIM and FOXO1, and reduced mRNA level of BCL-2 (Supplementary Figure 10B) when compared with WT mouse livers. Collectively, when compared with WT mouse livers, FAM3A^−/−^ mouse livers had decreased BCL-2/BAX mRNA and protein ratios. Rosiglitazone increased BCL-2/BAX ratio in WT mouse livers, but not in FAM3A^−/−^ mouse livers (Supplementary Figure 10B, Figure 4C). Rosiglitazone pretreatment increased cytosolic IκBα protein levels with a decrease in nuclear NF-κB in WT mouse livers (Figure 4D). Although FAM3A^−/−^ mouse livers had similar cytosolic IκBα protein level as WT mouse livers, they had significantly elevated nuclear NF-κB expression, which was not significantly affected by rosiglitazone pretreatment (Figure 4D). In support, FAM3A^−/−^ mouse livers had elevated mRNA levels of IL-1α and MCP-1 as WT mouse livers, which were not repressed by rosiglitazone (Supplementary Figure 10C). The expression levels of biomarkers for immune cells including CD3 (T cell marker), CD16 (Natural killer cell marker) and F4/80 (Macrophages) were increased in IRI FAM3A^−/−^ mouse livers, and not reversed by rosiglitazone treatment. In contrast, rosiglitazone reduced these biomarkers in WT IRI mouse livers (Supplementary Figure 11A-11B). The promoter region of phospholipid transfer protein (PLTP) contains several PPAR-responsive element (PPRE), and it is a target gene of peroxisome proliferator-activated receptors [20]. Rosiglitazone similarly upregulated PPARγ and PLTP expression in both WT and FAM3A^−/−^ mouse livers (Figure 4C, Supplementary Figure 10A, Supplementary Figure 11A), suggesting that FAM3A deficiency didn't affect the activation of PPARγ on other target genes. Overall, these findings strongly revealed that FAM3A-deficiency exaggerated liver IRI. Importantly, the protective effects of PPARγ activation on liver IRI was abolished in FAM3A^−/−^ mice.

### Rosiglitazone upregulated FAM3A to activate Akt and promote nuclear exclusion of FOXO1 in hepatocytes

**Figure 5:**
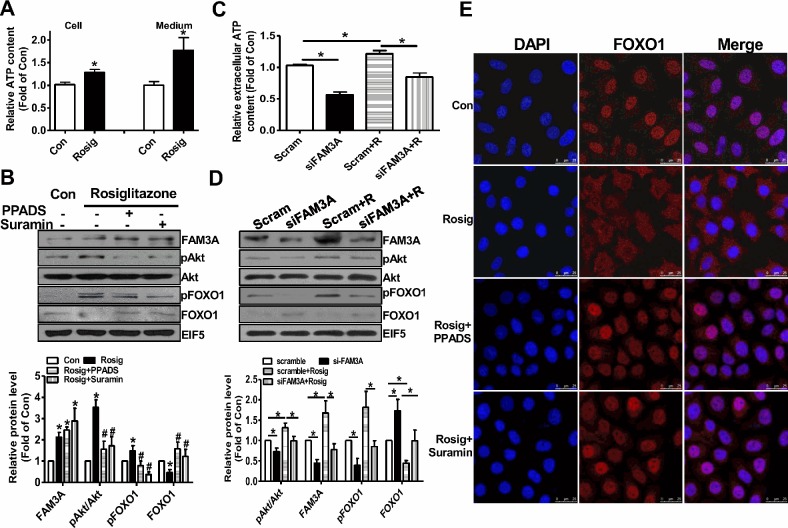
Rosiglitazone promoted Akt phosphorylation and nuclear exclusion of FOXO1 via FAM3A-ATP pathway in HepG2 cells The cell were treated with rosiglitazone for 36 hours, and then treated with PPADS or suramin for 1 hour before being performed for analysis. **(A)** Rosiglitazone pretreatment elevated cellular and extracellular ATP levels in HepG2 cells. **(B)** Inhibition of P2 receptors repressed rosiglitazone-induced phosphorylation of Akt and FOXO1. Representative gel images were shown in upper panel, and quantitative data shown in lower panel. N=5, *p<0.05 versus control cells; #p<0.05 versus rosiglitazone-treated cells. **(C)** Silencing of FAM3A inhibited rosiglitazone-induced elevation in extracellular ATP level. **(D)** Silencing of FAM3A inhibited rosiglitazone-induced Akt and FOXO1 phosphorylation. Representative gel images were shown upper panel, and quantitative data in lower panel. N=5, *p<0.05 between indicated groups. Scam, cell treated scrambled siRNA; siFAM3A, cells treated with siFAM3A; Scram+R, scrambled siRNA-transfected cells treated with rosiglitazone; siFAM3A+R, siFAM3A-transfected cells treated with rosiglitazone. **(E)** Inhibition of P2 receptors blocked rosiglitazone-induced nuclear exclusion of FOXO1. The images shown here were the representatives of at least 3 independent experiments.

**Figure 6:**
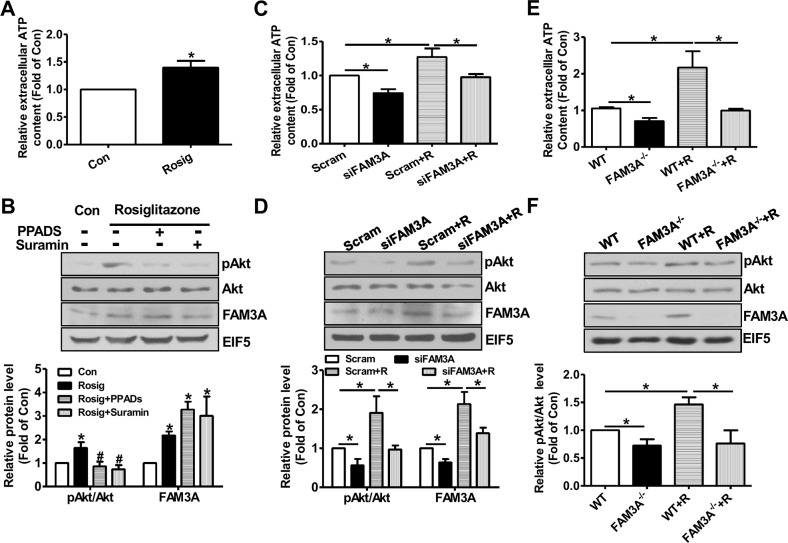
Knockdown or deficiency of FAM3A blunted rosiglitazone-induced Akt activation in primary mouse hepatocytes Primary mouse hepatocytes were treated with rosiglitazone for 36 hours, and then treated with PPADS or suramin for 1 hour before being performed for analysis. **(A)** Rosiglitazone treatment increased extracellular ATP level. **(B)** Rosiglitazone-induced Akt activation was inhibited by P2 receptor antagonists. Representative gel images were shown in upper panel, and quantitative data in lower panel. **(C)** Silencing of FAM3A inhibited rosiglitazone-induced increase in extracellular ATP level. **(D)** Silencing of FAM3A inhibited rosiglitazone-induced Akt activation. Representative gel images were shown in upper panel, and quantitative data in lower panel. **(E)** Deficiency of FAM3A abolished rosiglitazone-induced increase in extracellular ATP level. **(F)** Deficiency of FAM3A abolished rosiglitazone-induced Akt activation. Representative gel images were shown in upper panel, and quantitative data in lower panel. R, rosiglitazone. N=5, *p<0.05 between indicated two groups; ns, *no statistically* significant *difference* between indicated groups.

To further determine whether the beneficial effects of PPARγ activation on modulation of Akt and FOXO1 activities is mediated by FAM3A-ATP pathway, hepatocytes were pretreated with rosiglitazone for 36 hours, and then treated with inhibitors of purinergic *receptor 2* (P2 receptors) for 1 hour before the analyses of pAkt and pFOXO1 levels. Rosiglitazone treatment significantly elevated cellular and extracellular ATP levels in HepG2 cells (Figure 5A). Rosiglitazone treatment increased the protein levels of FAM3A, pAkt and pFOXO1 with a decrease in FOXO1 protein level (Figure 5B). Moreover, rosiglitazone-induced increases in pAkt and pFOXO1 levels were inhibited by P2 receptors PPADS and suramin with an increase in FOXO1 protein level (Figure 5B). To further confirm the role of FAM3A in rosiglitazone-induced Akt activation and FOXO1 inactivation, FAM3A expression was first knockdown by siRNA transfection, and followed by rosiglitazone treatment in HepG2 cells. Silencing of FAM3A reduced extracellular ATP content (Figure 5C), decreased cellular pAkt and pFOXO1 levels with increased cellular FOXO1 level (Figure 5D). Rosiglitazone-induced increase in extracellular ATP level, cellular pAkt and FOXO1 levels, and decrease in cellular FOXO1 level were reversed after FAM3A silencing in HepG2 cells (Figure 5D). In support, rosiglitazone treatment induced nuclear exclusion of FOXO1, which was blocked by inhibiting P2 receptors (Figure 5E) in HepG2 cells. In primary cultured mouse hepatocytes, rosiglitazone treatment elevated extracellular ATP level, FAM3A expression and pAkt level. Rosiglitazone-induced Akt activation was also blocked by P2 receptors (Figure 6A–6B). Silencing of FAM3A reduced extracellular ATP level and cellular pAkt level, and inhibited rosiglitazone-induced Akt activation in primary mouse hepatocytes (Figure 6C–6D). In support, FAM3A-deficient mouse hepatocytes exhibited lower extracellular ATP level and cellular pAkt level than WT mouse hepatocytes (Figure 6E–6F). Rosiglitazone failed to elevate extracellular ATP content and cellular pAkt level in FAM3A-deficienct hepatocytes (Figure 6E–6F). Overall, these findings revealed that the effects of PPARγ agonist on Akt activation and FOXO1 repression were dependent on FAM3A-ATP pathway.

### FAM3A overexpression protected, whereas FAM3A deficiency exaggerated oxidative-induced hepatocyte death

**Figure 7:**
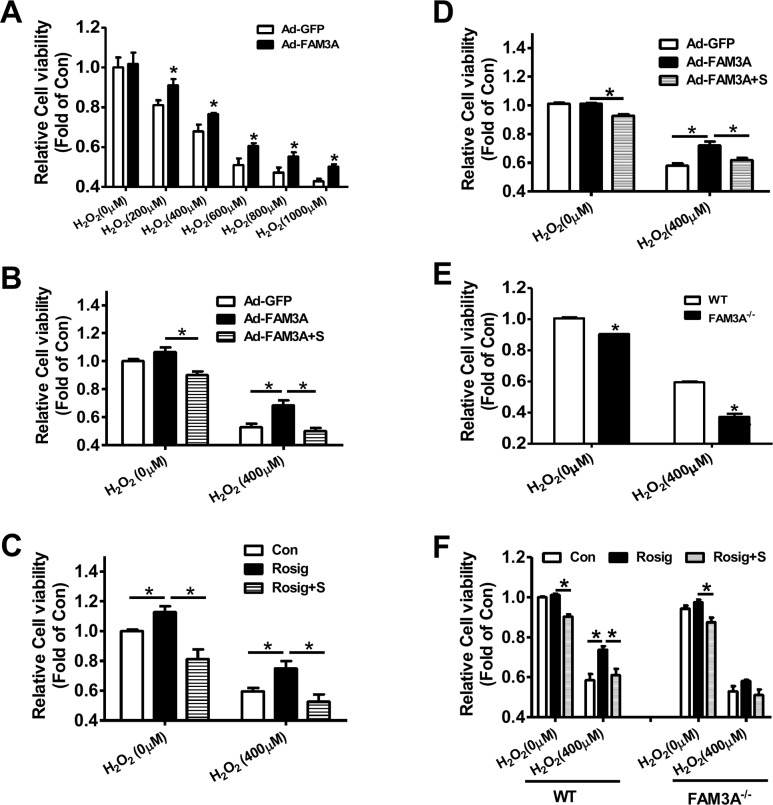
Deficiency of FAM3A exaggerated oxidative stress-induced cell death of hepatocytes **(A)** FAM3A overexpression protected against H_2_O_2_-induced cell death of HepG2 cells. **(B)** FAM3A's protection in H_2_O_2_-induced cell death was abolished by inhibiting P2 receptors in HepG2 cells. **(C)** Rosiglitazone's protection in H_2_O_2_-induced cell death was abolished by inhibiting P2 receptor in HepG2 cells. **(D)** FAM3A overexpression protected against H_2_O_2_-induced cell death of primary mouse hepatocytes. **(E)** FAM3A deficiency exaggerated H_2_O_2_-induced cell death of primary mouse hepatocytes. **(F)** Rosiglitazone's protection in H_2_O_2_-induced cell death was blunted in FAM3A-deficient mouse hepatocytes. S, in the presence of suramin. N=5, *p<0.05 between indicated two groups.

**Figure 8:**
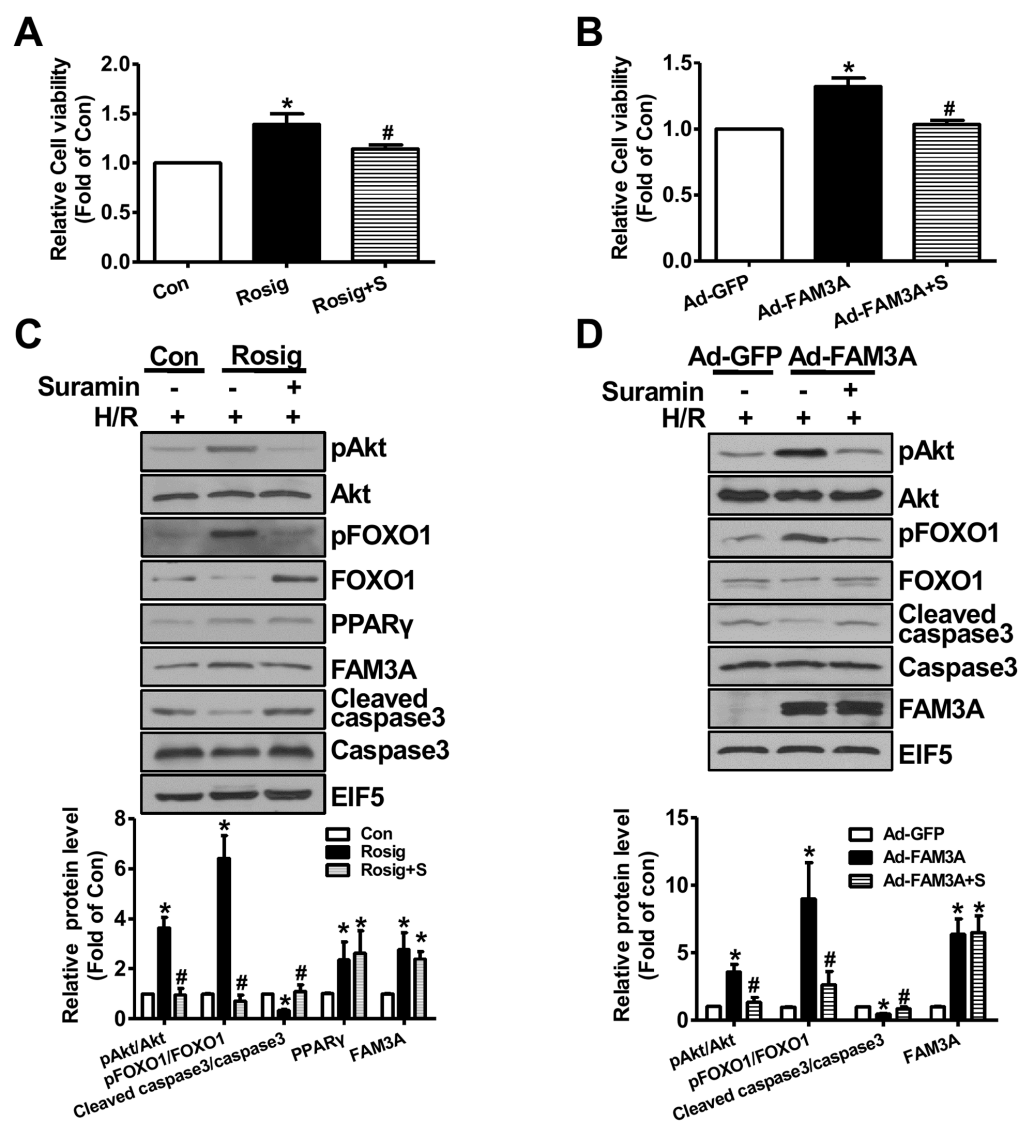
FAM3A activation or overexpression protected mouse hepatocyte against hypoxia/reoxygenation (H/R)-induced death Mouse hepatocytes were treated with rosiglitazone or Ad-FAM3A for 24 hours in the absence or presence of suramin, and then exposed to hypoxia for 4 hours, followed by 6 hours of reoxygenation. **(A-B)** Rosiglitazone treatment **(A)** or FAM3A overexpression **(B)** protected mouse hepatocytes against H/R-induced death. **(C-D)** Rosiglitazone treatment **(C)** or FAM3A overexpression **(C)** activated Akt and repressed caspase 3 activity in H/R-treated mouse hepatocytes. Rosig+S, hepatocytes treated with rosiglitazone in the presence of suramin; Ad-FAM3A+S, hepatocytes infected with Ad-FAM3A in the presence of suramin. N=3-5, *p<0.05 versus control cells; #p<0.05 versus rosiglitazone- or Ad-FAM3A-treated cells.

In HepG2 cells, H_2_O_2_ upregulated PPARγ and FAM3A expression (Supplementary Figure 12A-12B), which supported the observation that their expression were increased in IRI livers. FAM3A overexpression protected HepG2 cells against H_2_O_2_-induced death (Figure 7A), and the protective effect was abolished by inhibiting P2 receptors using suramin (Figure 7B). Rosiglitazone also exerted protective effects on H_2_O_2_-induced cell death in HepG2 cells, which was blunted by inhibiting P2 receptors (Figure 7C). In support, flow cytometry analyses revealed that rosiglitazone also protected mouse primary hepatocyte *against* H_2_O_2_-induced death (Supplementary Figure 13A-13B). Similarly, FAM3A overexpression protected against primary mouse hepatocyte death induced by H_2_O_2_, but was reversed by inhibiting P2 receptors (Figure 7D). In contrast, deficiency of FAM3A exaggerated primary mouse hepatocyte death induced by H_2_O_2_ (Figure 7E). Rosiglitazone's protection in mouse hepatocyte death induced by H_2_O_2_ was also blunted by inhibiting P2 receptors. Importantly, rosiglitazone's protective effect was almost abolished in FAM3A-deficient mouse hepatocytes (Figure 7F). In the basal condition without H_2_O_2_treatment, suramin treatment also reduced the cell viability in HepG2 cells and primary mouse hepatocytes (Figure 7B, 7C, 7D, 7F). Hypoxia/reoxygenation (H/R) experiment was further performed to validate the protective role of FAM3A in cultured mouse hepatocytes. After 4-hour hypoxia, mouse hepatocytes exhibited significant death in a time-dependent manner after reoxygenation (Supplementary Figure 13C). After H/R, the protein levels of PPARγ, FAM3A, FOXO1 and active caspase 3 were significantly increased (Supplementary Figure 13D). Rosiglitazone treatment or FAM3A overexpression protected mouse hepatocyte against H/R-induced death, but the protective effects were abolished by P2 receptor inhibition (Figure 8A–8B). In H/R treated mouse hepatocytes, rosiglitazone treatment or FAM3A overexpression activated Akt, and repressed FOXO1 and caspase activities, which were reversed by inhibiting P2 receptor (Figure 8C–8D). Collectively, these results suggested that FAM3A-ATP-P2 receptor-Akt pathways are important for the survival of hepatocytes under oxidative stress conditions.

## DISCUSSION

In the current study, we revealed that FAM3A exerts beneficial effects against liver IRI by activating ATP-Akt signaling pathways, repressing inflammation and attenuating oxidative stress. These novel findings greatly extend the understanding of FAM3A's biological functions beyond its roles in the regulation of glucose and lipid metabolism in the liver. Akt is an important survival molecule by targeting several proteins including FOXO1, BAX and BAD [17, 21, 22]. FOXO1 has been recognized as an apoptotic transcriptor, and its activation triggers apoptosis in various cell types by promoting the expression of several proapoptotic genes in response to stress [23–25]. BIM is a pro-apoptotic member of the B-cell CLL/lymphoma 2 (BCL-2) family of proteins, and upregulated BIM expression triggered apoptotic process in many cell types [26]. It had been reported that BIM is a target gene of FOXO1, and it mediates FOXO1's apoptotic effects in various studies under stress conditions [27–29]. In our study, we found that FAM3A knockdown or deficiency increases FOXO1 activity and BIM expression, whereas FAM3A upregulation represses FOXO1 activity and BIM expression in IRI livers, revealing that repression of FOXO1-BIM axis plays an important role in FAM3A's protection in liver IRI. In support, one recent study revealed that inhibition of FOXO1 activity is associated with attenuated liver IRI after PTEN silencing [17]. Beyond the repression of FOXO1 activity, FAM3A-induced Akt activation also exerts beneficial effects on liver IRI by increasing BCL-2/BAX ratio [30]. FAM3A knockdown or deficiency significantly reduces Akt activity, and decreases BCL-2/BAX protein ratio in IRI liver. These findings provide the clear and direct mechanism for explaining the deleterious effect of ATP depletion in liver IRI observed in previous studies [31]. It should be noted that the roles of PPARγ activation on regulation of Akt activity in HepG2 cells remain controversial. It had been reported that PPARγ activation increased Akt activity in HepG2 cells [13, 32–34], whereas other reports also suggested that PPARγ activation is associated with repressed Akt activity in HepG2 cells [35, 36]. These controversial findings suggested that further studies are still needed to clarify the distinct role of PPARγ on regulation of Akt activity in HepG2 cells or other hepatocellular carcinoma cell lines in various stress conditions.

Activation of nuclear factor κB (NF-κB) plays an important role in liver IRI by promoting the expression of proinflammatory cytokines [3]. Although it had been previously reported that Akt phosphorylates and activates IκB kinase (IKK) to induce NF-κB activation [37], the current study revealed that FAM3A knockdown or deficiency increases nuclear NF-κB fraction and inflammatory cytokine expression with repressed Akt activity in mouse liver with IRI. In support, rosiglitazone pretreatment attenuates liver IRI with upregulated FAM3A expression, increased Akt activity and reduced nuclear NF-κB expression. It is likely that FAM3A decreases nuclear NF-κB expression by increasing the degradation of cytosolic IκBα, which binds to NF-κB and retains it in cytoplasm. So far, whether or not FAM3A represses NF-κB activity via Akt pathway in IRI liver still remains unclear. Increased ROS production and oxidative stress play important roles in hepatocyte death during liver IRI. *In vivo*, FAM3A knockdown or deficiency elevated the serum and hepatic levels of oxidative stress marker MDA. Consistently, FAM3A deficiency decreased serum and hepatic T-AOC and SOD activity. Rosiglitazone pretreatment attenuated serum and hepatic oxidative stress in wild type mice, but not in FAM3A^−/−^ mice. In cultured hepatocytes, FAM3A overexpression or activation by rosiglitazone protects against, whereas FAM3A deficiency exaggerates oxidative-stress-induced cell death. FAM3A overexpression also represses ROS production stimulated by free fatty acids in cultured hepatocytes (data not shown). Overall, these findings revealed that FAM3A attenuates oxidative stress in liver IRI. One recent study revealed that FAM3A overexpression protects HT22 cells against hydrogen peroxide-induced oxidat cell death via activation of PI3K-Akt pathway [38]. Activation and recruitment of immune cells including macrophages also play important roles in liver IRI [39]. FAM3A deficiency increased the expression of markers for immune cells including CD3 [40], CD16 [41] and F4/80 [42], and rosiglitazone failed to inhibit their expression in IRI liver, suggesting that repression of immune cell activation also plays an important role in FAM3A's beneficial role in liver IRI. Collectively, FAM3A exerts protective effects against liver IRI via activation of Akt survival pathways. Moreover, repression of NF-κB activity and inflammation, attenuation of oxidative stress, inhibition of immune cell activation and reduction of NO production also contribute to FAM3A's beneficial effects on liver IRI.

Moreover, the current study provided strong *in vivo* and *in vitro* evidences that the beneficial effects of PPARγ activation on liver IRI are dependent on FAM3A-ATP pathways. *In vivo*, PPARγ activation failed to protect against liver IRI in FAM3A-deficient mice. *In vitro*, rosiglitazone's beneficial effects on protecting against oxidative-induced hepatocyte death were also dependent on FAM3A-ATP pathways. In IRI liver, both PPARγ and FAM3A expression are increased. In cultured mouse hepatocytes, H_2_O_2_ also induces PPARγ and FAM3A expression (Supplementary Figure 12A-12B). These findings indicated that activation of PPARγ-FAM3A axis is a protective mechanism against liver IRI. Overall, the findings in the current study and other previous studies [6–10] strongly suggested that oral administration of PPARγ agonists at 1-3 days before liver surgery or liver transplantation to activate hepatic FAM3A-ATP-Akt pathway is an effective strategy for attenuating human liver IRI.

In summary, FAM3A protects against liver IRI via activation of ATP-PI3K-Akt survival pathways, repression of NF-κB activation and attenuation of oxidative stress in hepatocytes. As a direct target gene of PPARγ, FAM3A mediates the protective effects of PPARγ agonists on live IRI.

## MATERIALS AND METHODS

### Animals

Male 8–12 weeks old wild-type (WT) and FAM3A^−/−^ mice on a C57BL/6 background were used. FAM3A^−/−^ mice were generated in Cyagen Biosciences Inc (China) using TALEN technology. Mice were housed in standard animal laboratories with a temperature maintained at 24°C and an artificial 12-hour light-dark cycle, with food and water ad libitum. All animal care and experimental protocols complied with the Animal Management Rules of the Ministry of Health of the People's Republic of China and the guide for the Care and Use of the Laboratory Animals of the Peking University.

### Antibodies

Anti-FAM3A antibody was purchased from Sigma (SAB1102488). Anti-pAkt (phosphorylated at Ser473 site) and Akt antibodies were bought from CST. Other antibodies were obtained from Santa Cruz, CST or other commercial companies.

### Partial liver ischemia/reperfusion injury (IRI) mouse model

All mice were anesthetized with pentobarbital (65 mg/kg) by intraperitoneal injection. Protocol for 70% liver ischemia model was detailed in previous studies [42, 43]. The duration of liver ischemia was 60 min, followed by a reperfusion for 6 hours. Body temperature was maintained at 37°C using a thermoregulatory heating pad. The animals had free access to water and rat chow *ad libitum*. After 6 h of reperfusion, anesthetized animals were sacrificed, and liver tissue and serum were collected for analysis. Sham-operated groups underwent the same surgical procedure, except that the blood supply to the liver lobes was not interrupted. To evaluate the role of FAM3A in IRI, siFAM3A (2.5mg/kg, the mixture of 3 sets of siRNAs) was injected to mice via the tail vein, the same dose of scrambled siRNA as control. Three days after siRNA injection, mice were subjected to IRI. To determine the role of PPARγ in liver IRI, mice were pretreated with methylcellulose or 10 mg/kg rosiglitazone dissolved in methylcellulose 3 times in 24 hours (8 hours interval) [9, 11]. 30 minutes after the final treatment, the mice were subjected to liver IRI.

### Primary mouse hepatocyte culture

Hepatocytes were isolated from mice by non-recirculating collagenase perfusion through the portal vein as previously described [14]. The isolated mouse hepatocytes were plated on dishes coated with rat collagen type I, confluence after plating was 80–90%, with hepatocyte viability of greater than 90% as assessed by Trypan blue exclusion. After plating, hepatocytes were cultured in RPMI 1640 containing 10% FBS at 37°C in 5% CO_2_ atmosphere.

### Cell culture and treatment

HepG2 cells were cultured in advanced DMEM medium (Invitrogen) supplemented with 10% fetal bovine serum, 2 mM L-glutamine, 100 units/ml penicillin, and 100units/ml streptomycin. All cells were cultured at 37°C in a humidified atmosphere consisting of 5% CO_2_ and 95% air. Rosiglitazone was purchased from Sigma and used at 25μM concentration for 36h. To study inhibitor assays, cells were treated with PPADS (P2X inhibitor, 50μM), suramin (P2X and P2Y inhibitor, 50μM) and LY294002 (PI3K inhibitor, 10μM) for 1 hour before being lysed for analysis. For FAM3A knockdown, siRNA against mouse or human FAM3A mRNA were purchased from Invitrogen. HepG2 or primary hepatocytes were transfected with 50nM siRNAs mixture (the mixture of 3-4 sets of siRNAs), the same concentration of scrambled siRNAs were used as negative control and incubated for 6 hours. After incubation, the cells were cultured in complete media containing 10% FBS in absence or presence of rosiglitazone (25μM, Sigma) for 36 hours before analysis. Cells were transfected with siRNAs, and subjected for further analysis after 36 hours. Cells were infected with 25 MOI Ad-GFP or Ad-FAM3A for 36 hours before further analysis. siRNA sequences against mouse and human FAM3A mRNA were provided in Supplementary Table 1.

### Western blotting

Proteins were extracted from mice liver and cells using lysis buffer containing fresh protease and phosphatase inhibitors. Cell lysates and homogenates were centrifuged at 12000rpm for 10 minutes at 4°C. Protein contents in the supernatant were quantified using BCA Protein Assay Kit. Protein samples were separated by SDS-PAGE and transferred to a nitrocellulose membrane. Immunoblotting was conducted using primary antibodies against target genes. After overnight incubation with primary antibody, membranes were washed and incubated with HRP-conjugated secondary antibodies and were detected using chemiluminescence kit. EIF5 or β-actin was analyzed using a rabbit polyclonal as loading control.

### Nuclear and cytoplasmic protein extraction

Nuclear cytoplasmic protein extracts were extracted from 50-100mg mouse liver according to the manufacturer's instructions using a nuclear extraction kit (Thermo, Prod#78833). The extracted cytosolic fraction and nuclear fraction were analyzed by Western blotting. β-actin and Histone H3 were used as loading control for cytosolic and nuclear fraction, respectively.

### RNA extraction and Real-time PCR

Total RNA was isolated from each 30 mg liver tissue by homogenization in 1ml TRIzol reagent (Invitrogen, USA) according to the manufacturer's instructions. RNA (3-5μg) was converted to cDNA using cDNA synthesis kit (Thermo scientific, USA) following the manufacturer's standard protocol. The protocol for real-time PCR analysis is as following: 95°C for 5 min, followed by 40 cycles at 95°C for 30s, 59°C for 30s, and 72°C for 30s. The Cycle threshold (Ct) values for the targets and GAPDH genes were provided by real-time PCR instrumentation. The comparative method 2^−ΔΔCt^ was used for the relative quantification of target gene transcription between the control and the treated groups [14]. The sequences for the primers used for real-time PCR are provided in Supplementary Table 2.

### HE staining and Immunohistochemistry

All tissues were fixed in 10% formalin immediately after surgical resection and embedded in paraffin. Liver tissues were cut to 5-μm sections on a microtome which was made into paraffin sections. The tissue sections were deparaffinated by immersion in xylene and rehydration, and then stained with hematoxylin-eosin (HE) and examined using light microscopy. For immunohistochemistry, sections were incubated with 3% hydrogen peroxide to block endogenous peroxidase activity. Tissue sections were blocked with 10% BSA for 1 h and incubated with primary antibodies at 4°C overnight. A 1:100 dilution of anti-FAM3A or anti-PPARγ antibody was used as the primary antibody.

### Confocal analysis of forkhead box O1 (FOXO1) nuclear exclusion

Cells were grown on coverslips as previously described [14], media was removed at 24 hours post treatment. All samples were rinsed with PBS, and then permeabilized with 0.2% Triton X-100/0.5% BSA in PBS for 10 minutes, followed by washing with PBS. The coverslips were blocked in 1% BSA for 30 minutes at 37°C. Blocking buffer was then removed, and coverslips were incubated with primary antibody anti-FOXO1 in blocking buffer at 4°C overnight. The primary antibody was then removed, and three 5-minute washes in PBS were performed. Followed, detected with goat anti-rabbit Alexa Fluor 594. After nuclear staining with DAPI, coverslips were mounted on glass slides using 70% glycerol in PBS. Mounted coverslips were imaged and cells were visualized by fluorescence microscopy using Confocal Laser Scanning Microscope.

### Cell survival assay

Cell viability was quantified using 3-(4,5-Dimethylthiazol-2-yl)2,5-diphenyl tetrazolium bromide (MTT). Cells were seeded at a density of 3 × 10^3^ cells/96-well plate, and then treated with H_2_O_2_ with different concentration (0μM, 200μM, 400μM, 600μM, 800μM, 1000μM) at 37°C, 5% CO_2_. For detecting the protective role of FAM3A, cells were infected with Ad-GFP or Ad-FAM3A for 3 hours, and then treated with H_2_O_2_ for 24 hours in the absence or presence of 50μM suramin. For rosiglitazone experiments, cells were treated with 25μM rosiglitazone in medium containing H_2_O_2_ with or without suramin for 24 hours. MTT assays were performed as detailed previously [15].

### Activity assay of ALT, AST and others

Blood samples were centrifuged for 10 minutes at 5000 rpm and serum samples were stored at -20°C until the measurement. The activities of serum alanine aminotransferase (ALT) and aspartate aminotransferase (AST) were determined in Department of Laboratory Medicine of Peking University Third Hospital. The superoxide dismutase (SOD), myeloperoxidase (MPO), methane dicarboxylic aldehyde (MDA) and total antioxidant capacity (T-AOC) in the supernatant were determined using assay kit (Nanjing Jiancheng Corp, China).

### ATP measurement

Liver tissues or cultured cells were lysed in ATP-Lite Assay Kit lysis buffer (Vigorous Biotechnology Beijing Co., Ltd). The medium of cultured cells was also collected for ATP determination. For determination of relative ATP level in the cells, the ATP content values (nmol) were first normalized to the protein mount (nmol/mg protein) in the same sample, and then normalized to the control values. For determination of relative ATP level in the medium, the absolute concentration was determined and normalized to the control value [14, 44].

### Hypoxia/reoxygenation (H/R) experiments

Primary hepatocytes were isolated and cultured for 6 hours. Hepatocytes were treated with rosiglitazone or Ad-FAM3A for 24 hours, and then subjected to 4 hours of hypoxia in the incubator with 5% CO_2_/95% N_2_. For reoxygenation, hepatocytes were removed from hypoxia incubator to incubate at 37°C with a 95% air/5% CO_2_ atmosphere for various time length before experimental assays.

### Statistical analysis

The results are presented as the mean ± SEM. Statistical significance of differences between groups was analyzed by t-test or by one-way analysis of variance (ANOVA) when more than two groups were compared. P values < 0.05 were considered as statistically significant.

## SUPPLEMENTARY MATERIALS FIGURES AND TABLES



## Abbreviations

FAM3: family with sequence similarity 3
FAM3A: family with sequence similarity 3 member A
PPARγ: peroxisome proliferator activated receptor gamma
Akt: Protein kinase B
I/R: ischemia/reperfusion
IRI: ischemia/reperfusion injury
AST: aspartate aminotransferase
ALT: alanine aminotransferase
SOD: superoxide dismutase
T-AOC: total antioxidant capacity
MDA: methane dicarboxylic aldehyde
MPO: myeloperoxidase
ATP: adenosine triphosphate
FOXO1: forkhead box O1
ROS: reactive oxygen species
EGF: epidermal growth factor
IGF-1: insulinlike growth factor-1
HE: hematoxylin-eosin
MTT: 3-(4,5-Dimethylthiazol-2-yl)2,5-diphenyl tetrazolium bromide
H/R: hypoxia/reoxygenation
NF-κB: nuclear factor kappa B
Bcl-2: B-cell lymphoma-2
GDH: glutamate dehydrogenase
NO: nitric oxide
PLTP: phospholipid transfer protein
PPRE: PPAR-responsive element
PTEN: phosphatase and tensin homolog
IKK: IκB kinase.
