# Supplementary material for: FAM3A mediates PPARγ's protection in liver ischemia-reperfusion injury by activating Akt survival pathway and repressing inflammation and oxidative stress
Source: Oncotarget. 2017 May 11;8(30):49882–96. doi: 10.18632/oncotarget.17805 (PMC5564815; doi:10.18632/oncotarget.17805)
Supplement: Supplementary file 1 [file oncotarget-08-49882-s001.pdf]

FAM3A mediates PPARγ's protection in liver ischemia-reperfusion injury by activating Akt survival pathway and repressing inflammation and oxidative stress

SUPPLEMENTARY MATERIALS

Cell apoptosis analysis

Apoptosis was detected using Annexin V-FITC/7-AAD staining. Briefly, mouse hepatocytes were treated with water, H_2O_2 (400μM), H_2O_2+Rosig for 24h. 1–2×10^6 cells were trypsinized using EDTA-free trypsin and centrifuged at 1000 rpm. The cells were collected and washed twice in PBS, and then labeled with 7-AAD and Annexin V-FITC in binding buffer for 15 min in the dark according to manufacturer's instructions. Analyses were performed with flow cytometry.

Mitochondria isolation

The isolation of mitochondrial and cytosolic fractions were performed according to the instructions for the Mitochondria/Cytosol Isolation Kit (C1260, Applygen Technologies Inc.). 30mg livers were homogenized in 1.5mL of ice-cold Mito-Cyto Buffer with a Dounce homogenizer. After centrifugation twice at 800g for 5 min at 4°C, the supernatant was collected, transferred to a fresh microcentrifuge tube, and centrifuged twice at 12,000g for 10 min at 4°C. The supernatant is cytosolic fraction. The pellet, which contained the mitochondria, was resuspended in 30μl Mito-CytoBuffer. The protein concentration was determined using the bicinchoninic acid method. β-actin was used as cytosolic marker, whereas ATP synthase β subunit (ATPSβ) was used as mitochondrial marker.

Measurement of nitric oxide

Briefly, 30-50mg liver tissue was homogenized in saline. After centrifugation twice at 1000rpm for 10 min at 4°C, the supernatant was collected. Nitric oxide (NO) level was determined according to the instructions for determination kit (A013-2, Nanjing Jiancheng Corp). Absorbance at 550 nm was immediately recorded and compared to the absorbance of a freshly prepared standard curve of sodium nitrite. Finally, nitric oxide content were normalized to total protein content.

Glutamate dehydrogenase (GDH) activity assay

GDH activity was performed according to the instructions for Glutamate dehydrogenase detection kit (ab02527, Abcam). Briefly, 50mg liver was homogenized in 200μl ice cold assay buffer, and then centrifuged at 13000g for 10min to remove insoluble content. The GDH activity was determined according to the instruction of kit. GDH activity was normalized to total protein content in the same sample.

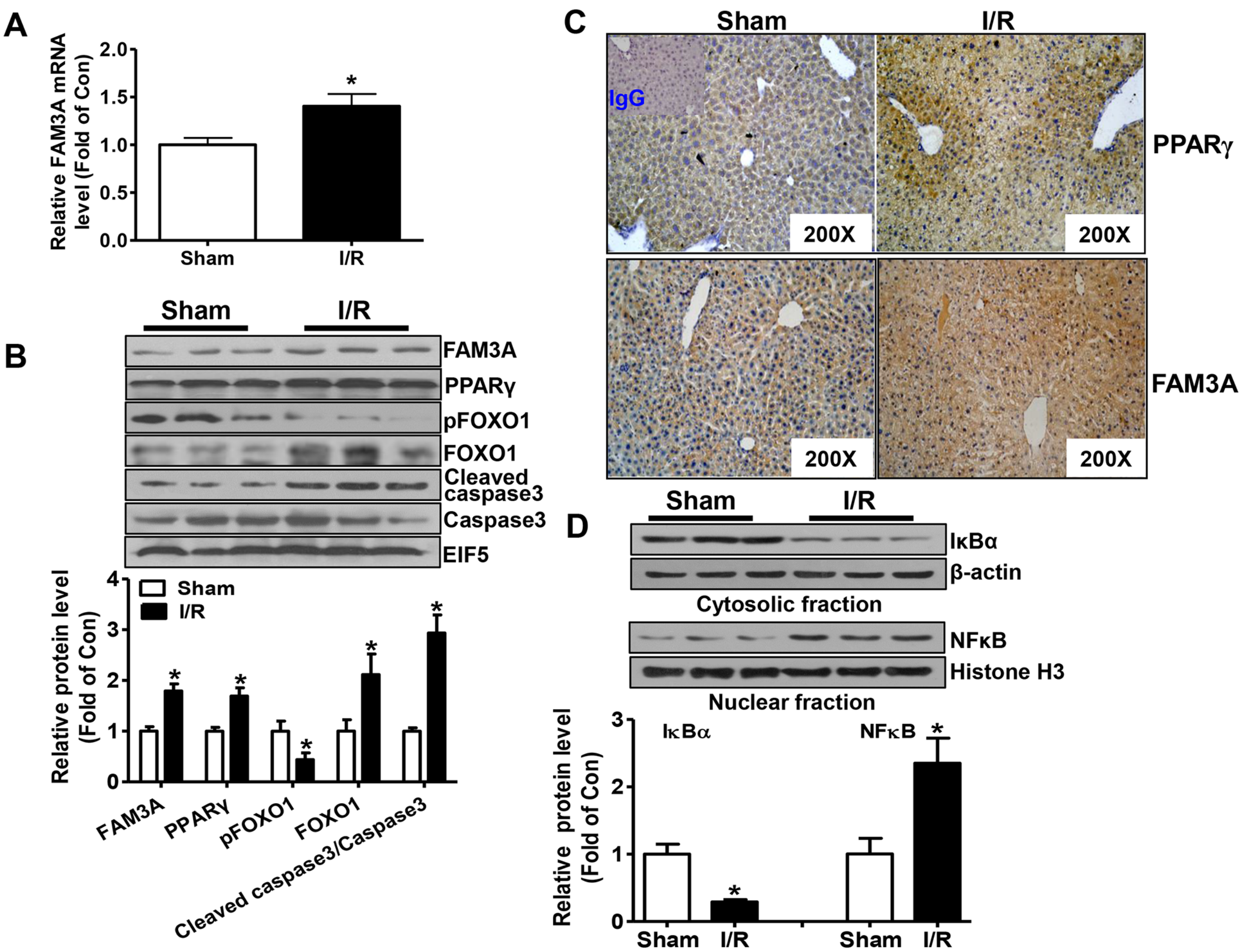

Supplementary Figure 1: FAM3A expression was increased in liver with IRI. (A) FAM3A mRNA level was increased in liver with IRI. **(B)** Western blotting assays of FAM3A expression in liver samples with IRI. Representative gel images were shown in upper panel, and quantitative data in lower panel. **(C)** Immunohistochemical staining assay of increased FAM3A expression in liver samples with IRI. **(D)** Nuclear distribution of NFκB in mouse livers with IRI. N=6-8, *p<0.05 versus sham group of mice.

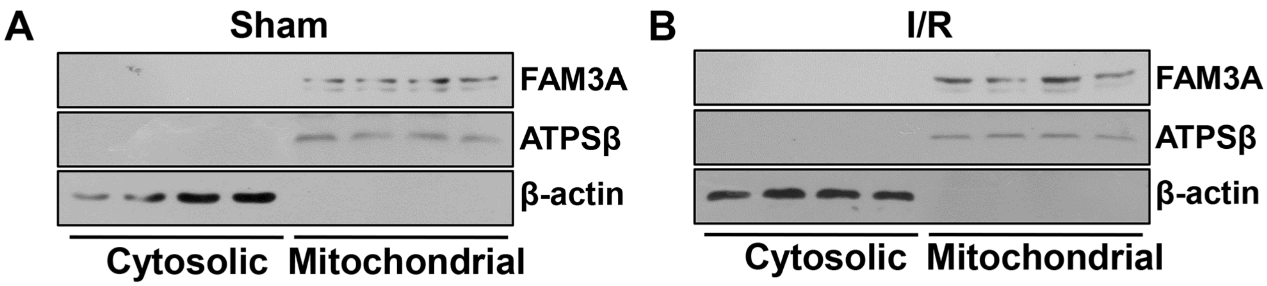

Supplementary Figure 2: FAM3A protein was located in mitochondria of control and IRI mouse livers. FAM3A protein is located in mitochondria of mouse livers with Sham or IRI. Sham, mice with sham; IRI, mice with liver IRI. ATPSβ, ATP synthase β subunit (mitochondrial marker).

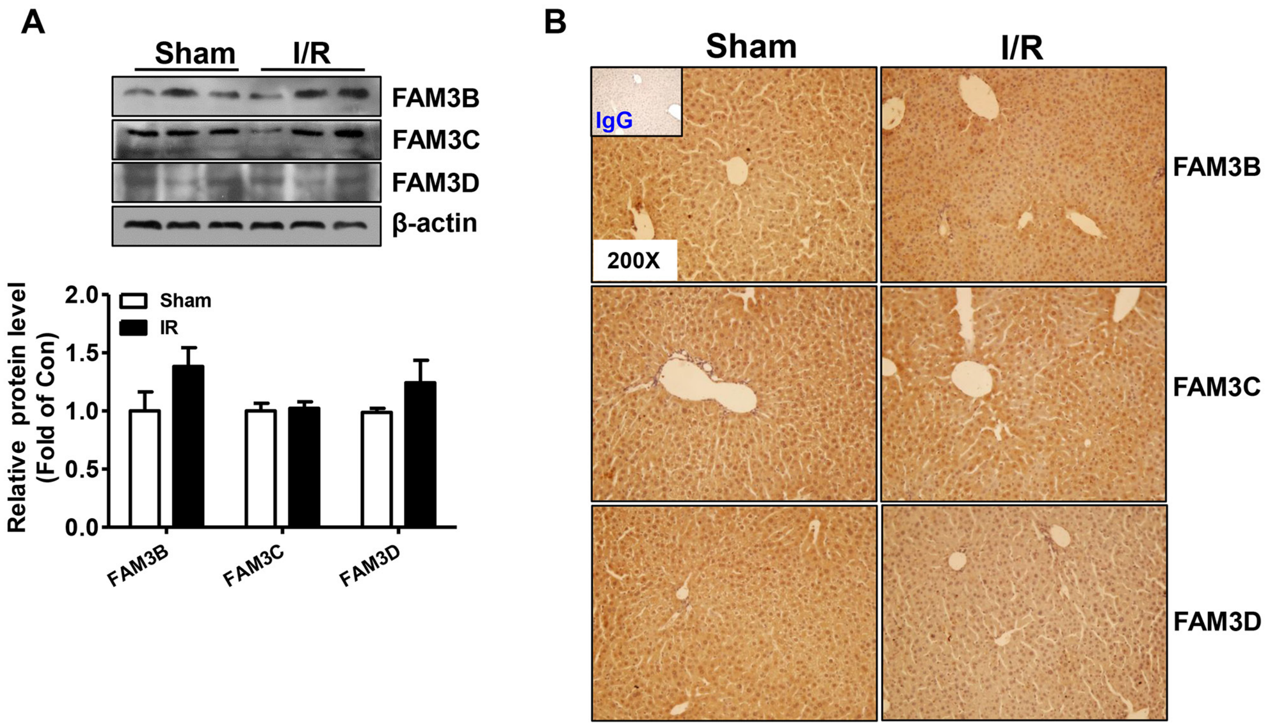

Supplementary Figure 3: The expression of FAM3B, FAM3C and FAM3D remained unchanged in IRI mouse livers. **(A)** Western blotting assays of FAM3B-D protein levels in IRI mouse livers. N=5, there was no significant difference between two groups. **(B)** Immunohistochemical staining of FAM3B-D proteins in IRI mouse livers. The images were the representatives of three mouse livers.

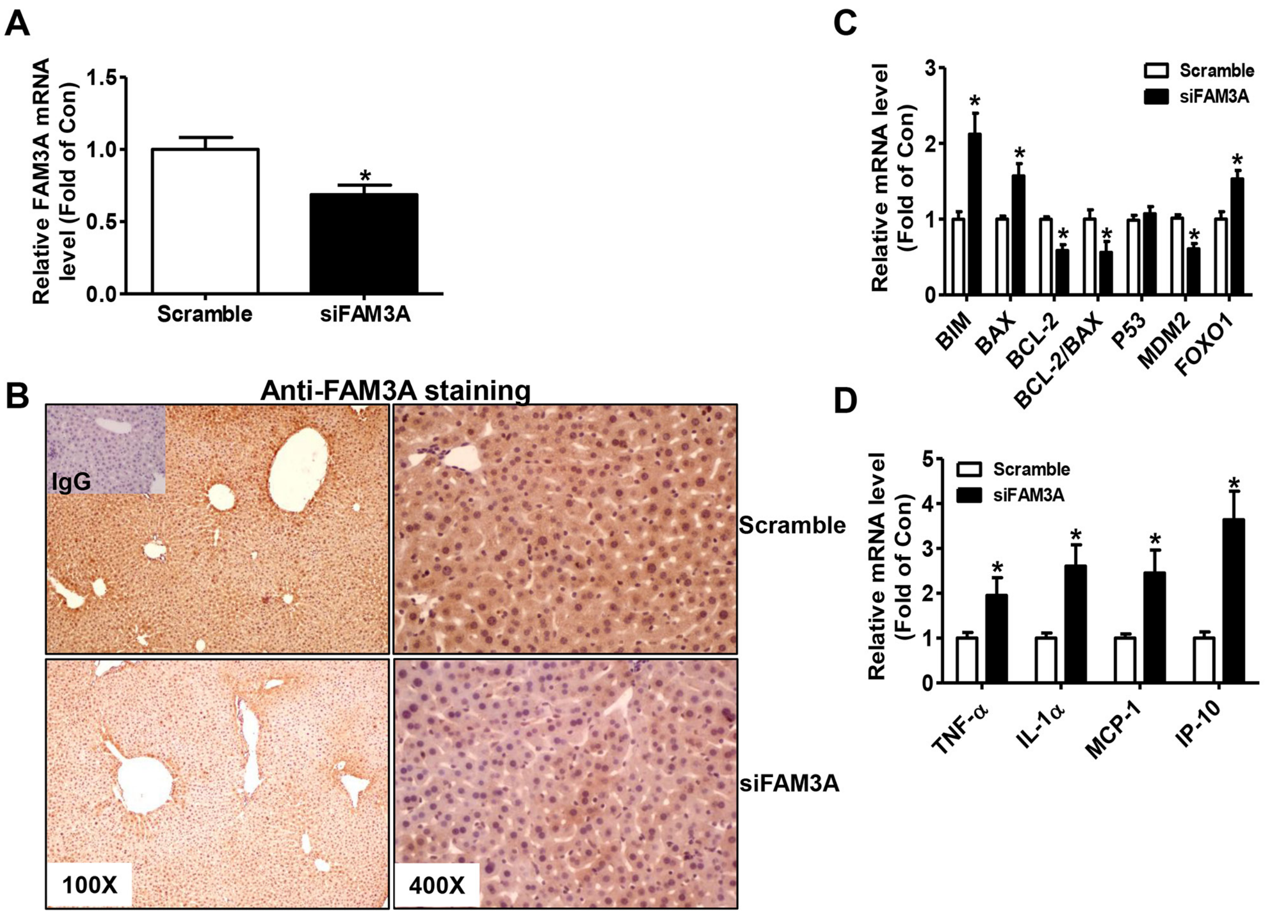

Supplementary Figure 4: Silencing of FAM3A on the mRNA expression of apoptotic genes and inflammatory cytokines in mouse liver with IRI. **(A)** siFAM3A treatment reduced FAM3A mRNA level in mouse livers with IRI. **(B)** Immunohistochemical staining assays of FAM3A expression in liver treated with siFAM3A. **(C)** Silencing of FAM3A on the mRNA expression of apoptotic genes in mouse liver with IRI. **(D)** Silencing of FAM3A on the mRNA expression of inflammatory cytokines in mouse liver with IRI. N=8, *$p<0.05$ versus sham group of mice.

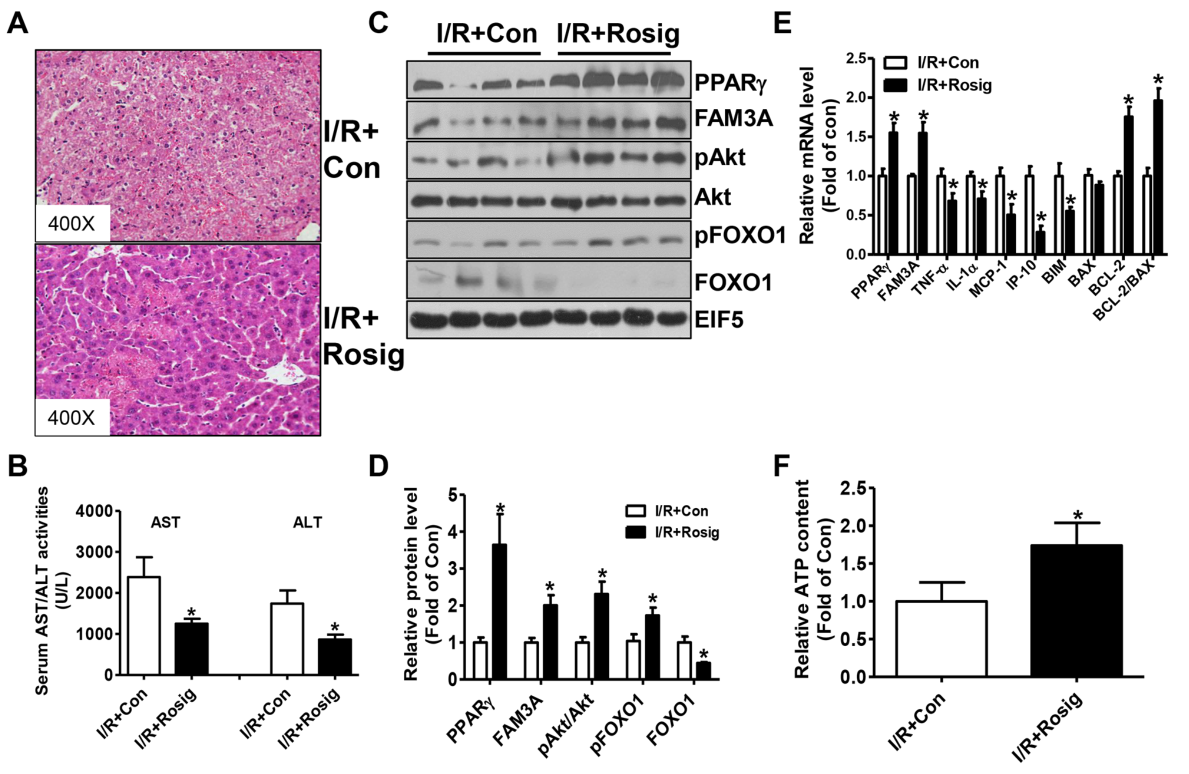

Supplementary Figure 5: Rosiglitazone pretreatment protected against liver IRI. **(A)** H.E staining of IRI liver samples pretreated with rosiglitazone. **(B)** Rosiglitazone pretreatment reduced serum AST and ALT activities after liver IRI. **(C-D)** Rosiglitazone pretreatment upregulated FAM3A expression in mouse liver with IRI. Representative gel images were shown in C, and quantitative data in **(D-E)** Rosiglitazone pretreatment repressed the mRNA levels of inflammatory cytokines and increased the mRNA level of anti-apoptotic gene in mouse liver with IRI. **(F)** Rosiglitazone pretreatment elevated cellular ATP content in mouse liver with IRI. N=6-8, *$p<0.05$ versus control group of mice.

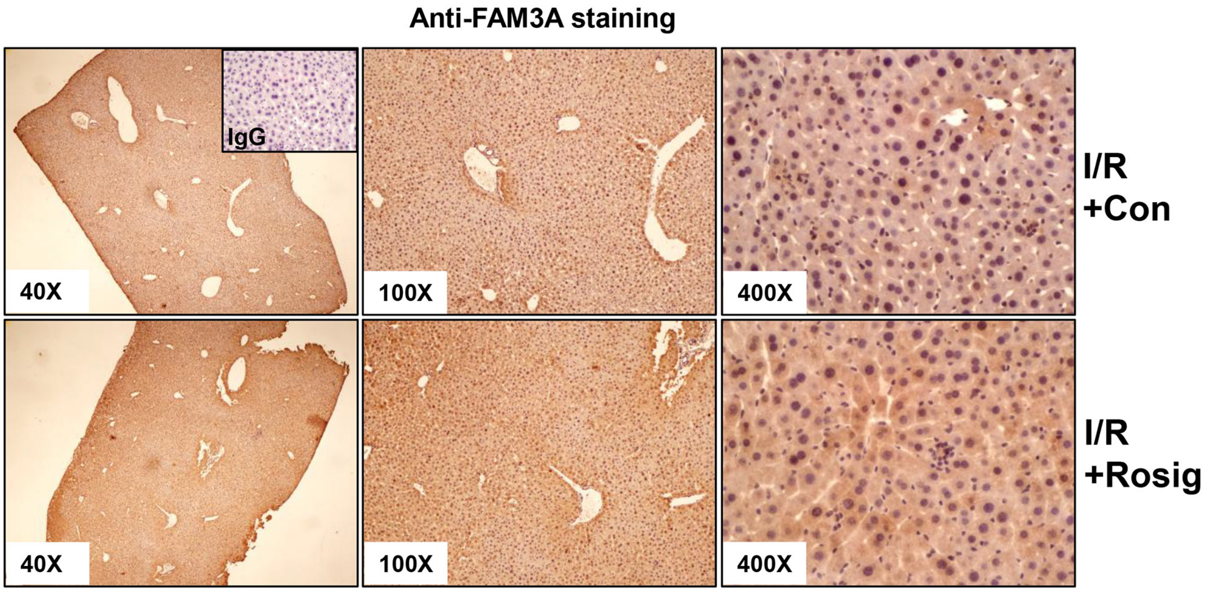

Supplementary Figure 6: Rosiglitazone pretreatment upregulated FAM3A protein level in mouse liver with IRI. The immunohistochemical staining images shown here were the representatives of at least 3 liver samples. IgG, negative control stained with anti-IgG antibodies.

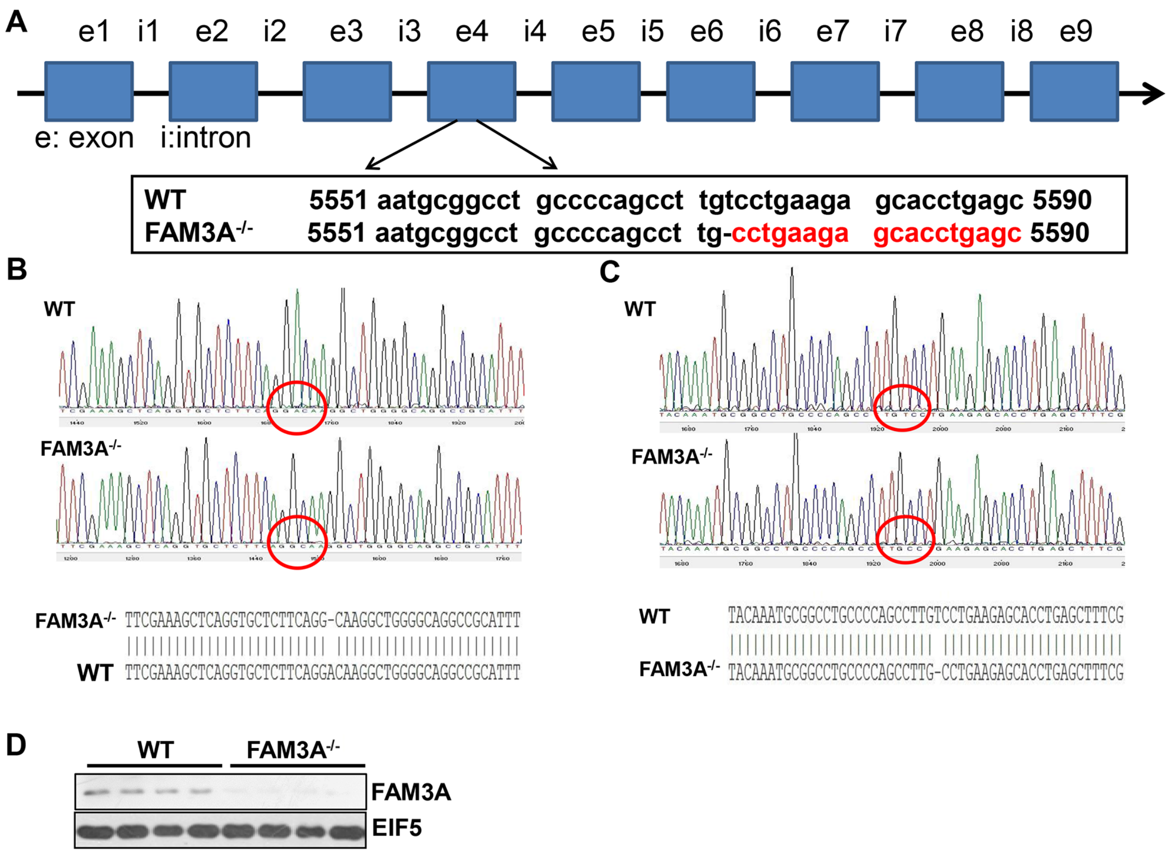

Supplementary Figure 7: Generation of FAM3A$^{-/-}$ mice using TALEN technology. FAM3A$^{-/-}$ mice were generated in Cyagen Biosciences Inc (China) using TALEN technology. **(A)** Schematic map of deletion of one nucleotide in exon 4 of mouse FAM3A gene. **(B)** Validation of FAM3A$^{-/-}$ mice by genomic DNA sequencing of tail samples. **(C)** Validation of FAM3A$^{-/-}$ mice by mRNA sequencing of liver samples. **(D)** FAM3A protein is deficient in FAM3A$^{-/-}$ mouse livers. WT, wild type mice; FAM3A$^{-/-}$, FAM3A-deficient mice; e1-e9 and i1-i8 represent exon 1-9 and intron 1-8 of mouse FAM3A gene, respectively.

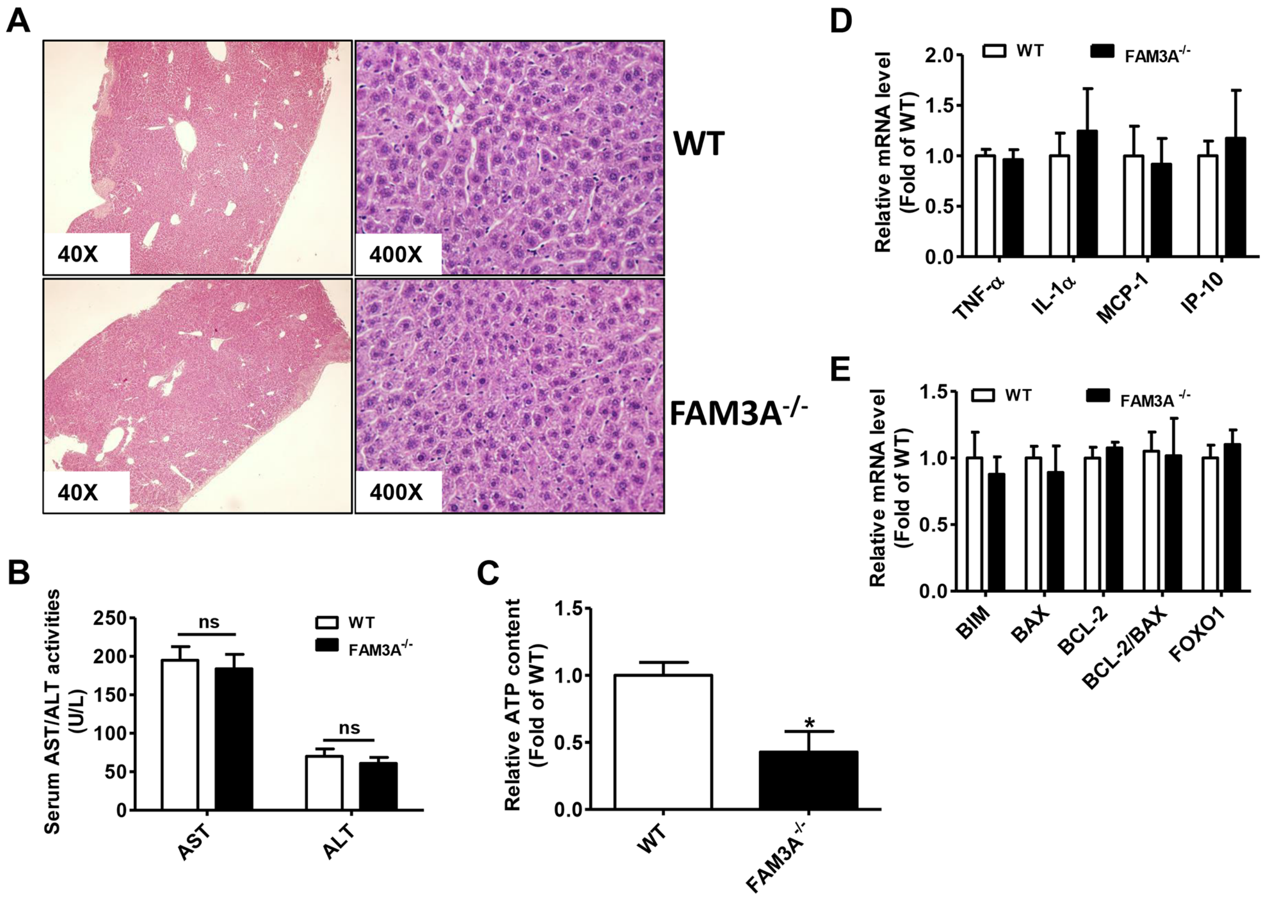

Supplementary Figure 8: FAM3A$^{-/-}$ mice exhibit normal liver structure in physiological condition. 8-12-week-old male FAM3A$^{-/-}$ mice were used in this study. A-B) In physiological condition, FAM3A$^{-/-}$ mice display normal liver structure **(A)** and serum AST/ALT activities **(B)** as WT mice. **(C)** FAM3A$^{-/-}$ mouse livers have lower ATP content than WT mouse livers in physiological condition. **(D-E)** FAM3A$^{-/-}$ mouse livers have similar mRNA levels of inflammatory cytokines and apoptotic genes in physiological condition. N=8, *p<0.05 versus WT mice.

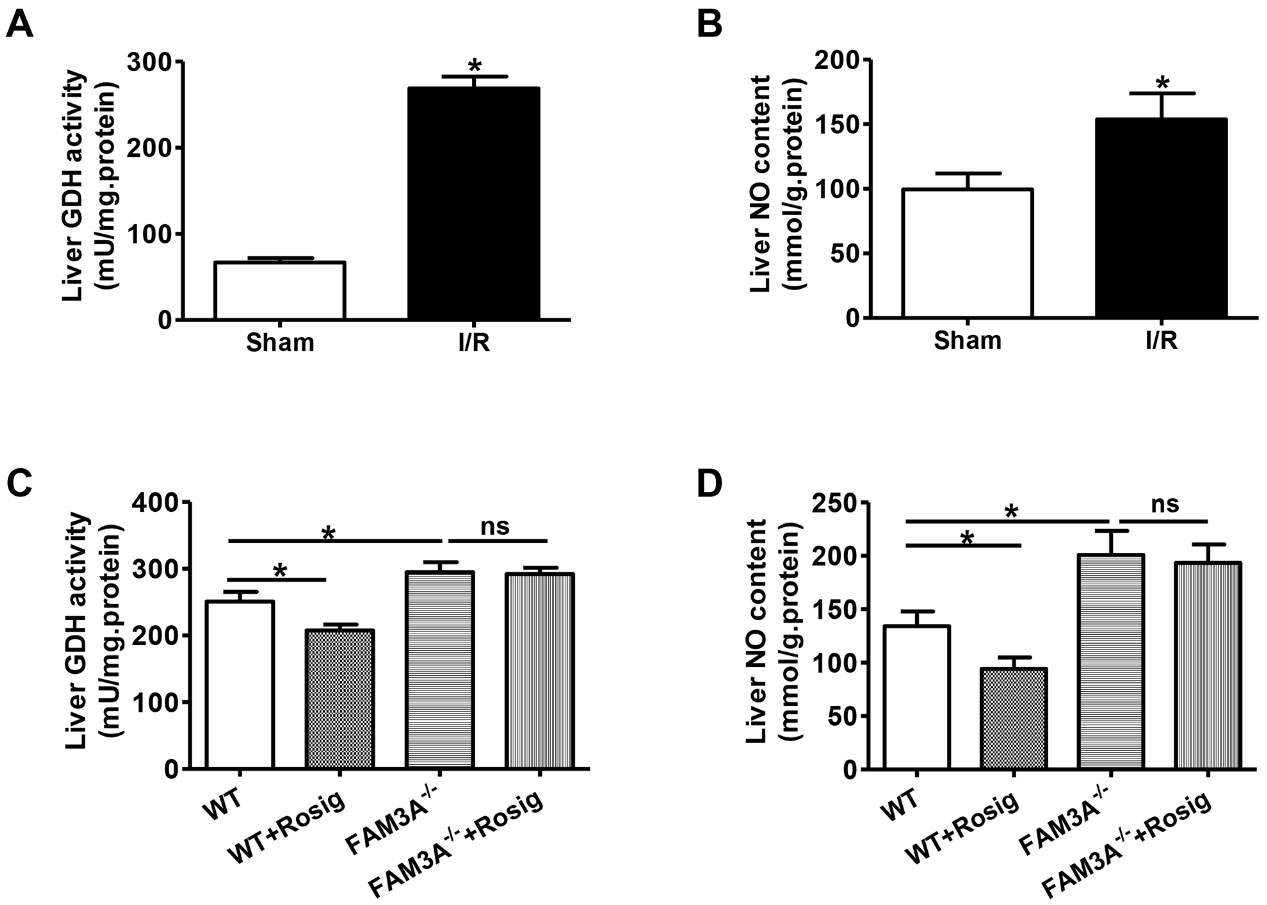

Supplementary Figure 9: FAM3A deficiency increased GDH activity and NO level in IRI livers. (A-B) GDH activity **(A)** and NO level **(B)** were increased in mouse livers after IRI. N=7, *p<0.05 versus sham mouse livers. **(C-D)** FAM3A deficiency increased GDH activity **(C)** and NO level **(D)** in mouse livers after IRI. GDH, glutamate dehydrogenase; NO, nitric oxide. N=6-8, *p<0.05 versus WT mice without rosiglitazone treatment.

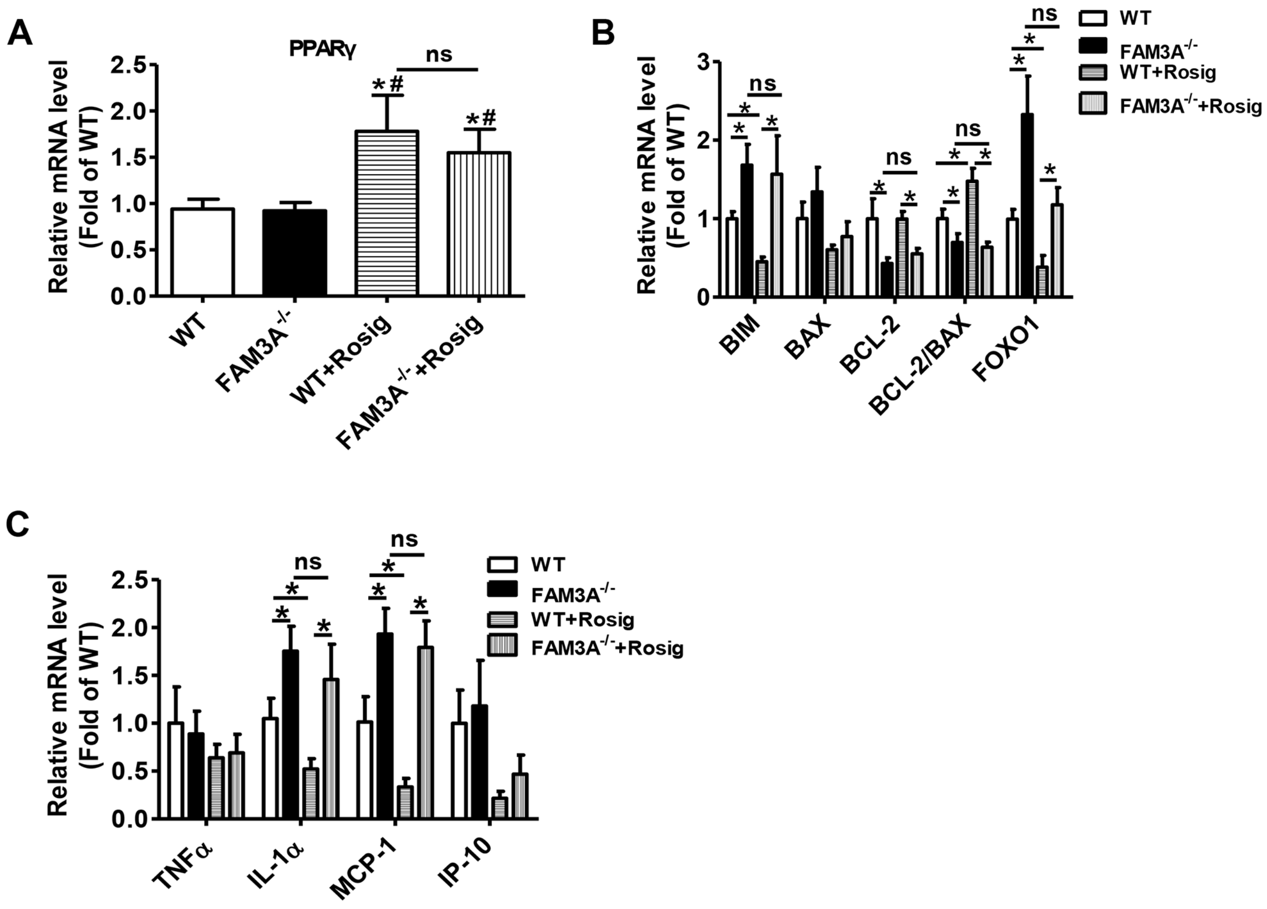

Supplementary Figure 10: Rosiglitazone pretreatment on mRNA levels of apoptotic genes and inflammatory cytokines in $FAM3A^{-/-}$ mouse livers. WT or $FAM3A^{-/-}$ mice were orally treated with rosiglitazone, and then subjected to liver IRI. **(A)** Rosiglitazone pretreatment increased PPARγ mRNA in $FAM3A^{-/-}$ mouse livers with IRI. **(B)** Rosiglitazone pretreatment on the mRNA levels of apoptotic and anti-apoptotic genes in mouse livers with IRI. **(C)** Rosiglitazone pretreatment on the mRNA levels of inflammatory cytokines in mouse livers with IRI. N=8, *$p<0.05$ between indicated two groups; ns, no statistically significant difference between indicated groups.

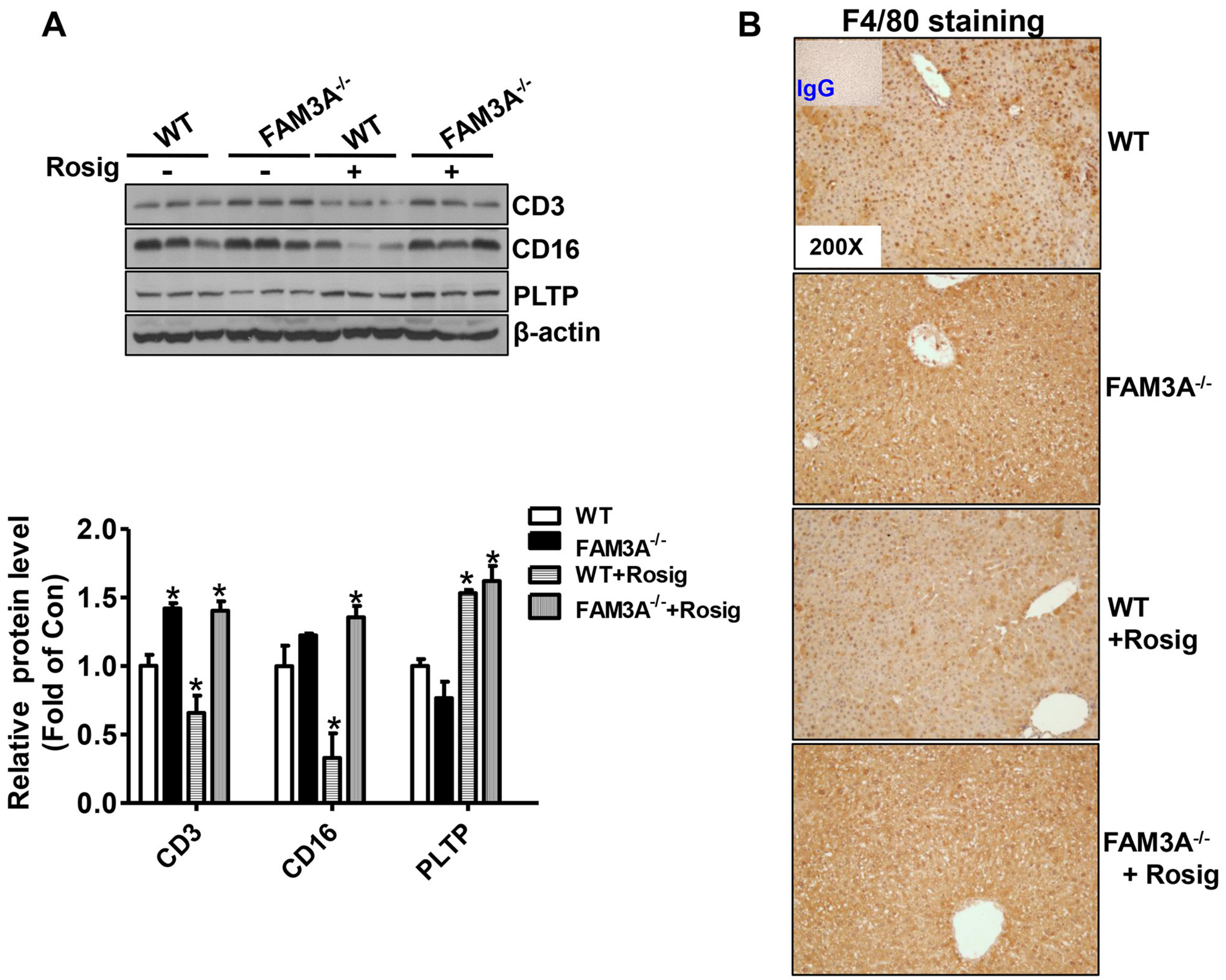

Supplementary Figure 11: FAM3A deficiency increased the expression of biomarkers for immune cells in IRI livers. **(A)** Western blotting assays of protein levels in IRI mouse livers. N=6, *$p<0.05$ versus WT mice without rosiglitazone treatment. **(B)** Immunohistochemical staining of F4/80 protein in IRI mouse livers. The images were the representatives of three mouse livers.

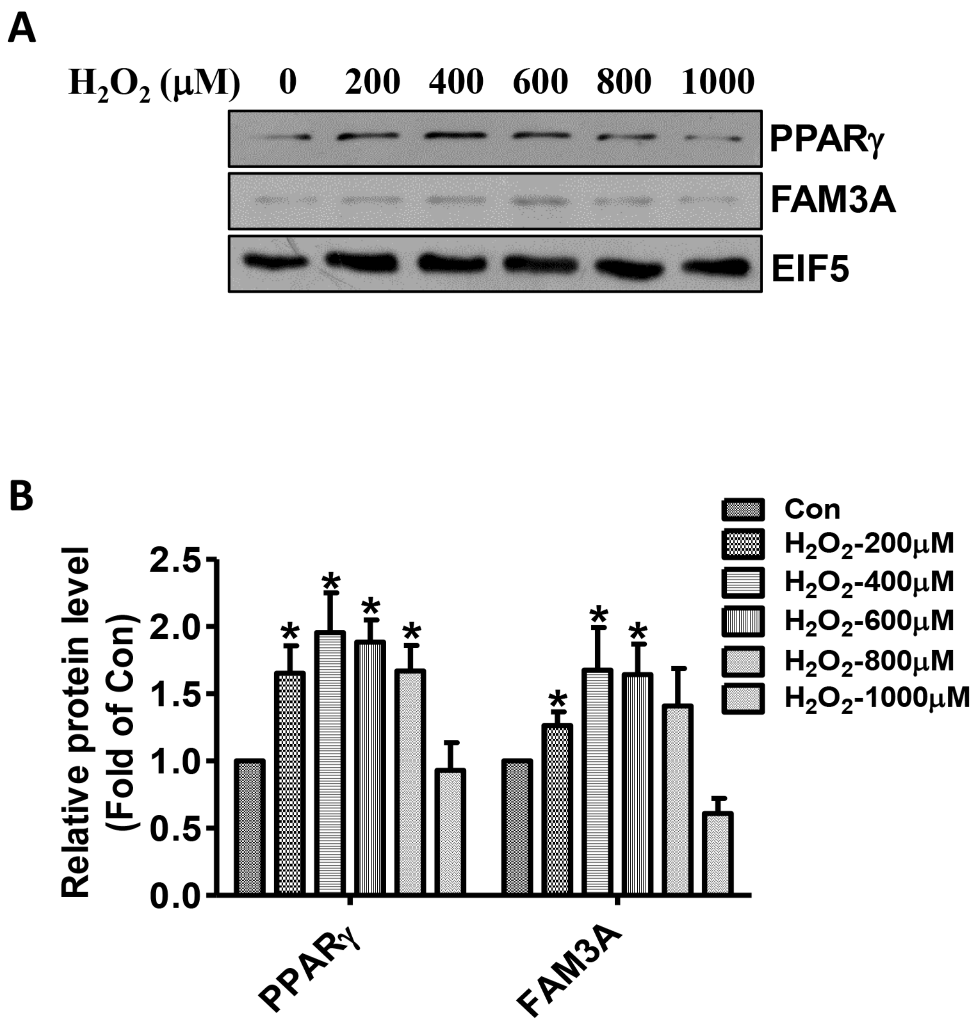

Supplementary Figure 12: Oxidative stress activated PPARγ and FAM3A expression in primary mouse hepatocytes. Primary mouse hepatocytes cells were treated with various concentration of H_2O_2 for 24 hours, and then PPARγ and FAM3A protein levels were analyzed. **(A-B)** H_2O_2 induced PPARγ and FAM3A expression Representative gel images were shown in A, and quantitative data in B. N=4, *p<0.05 versus control cells.

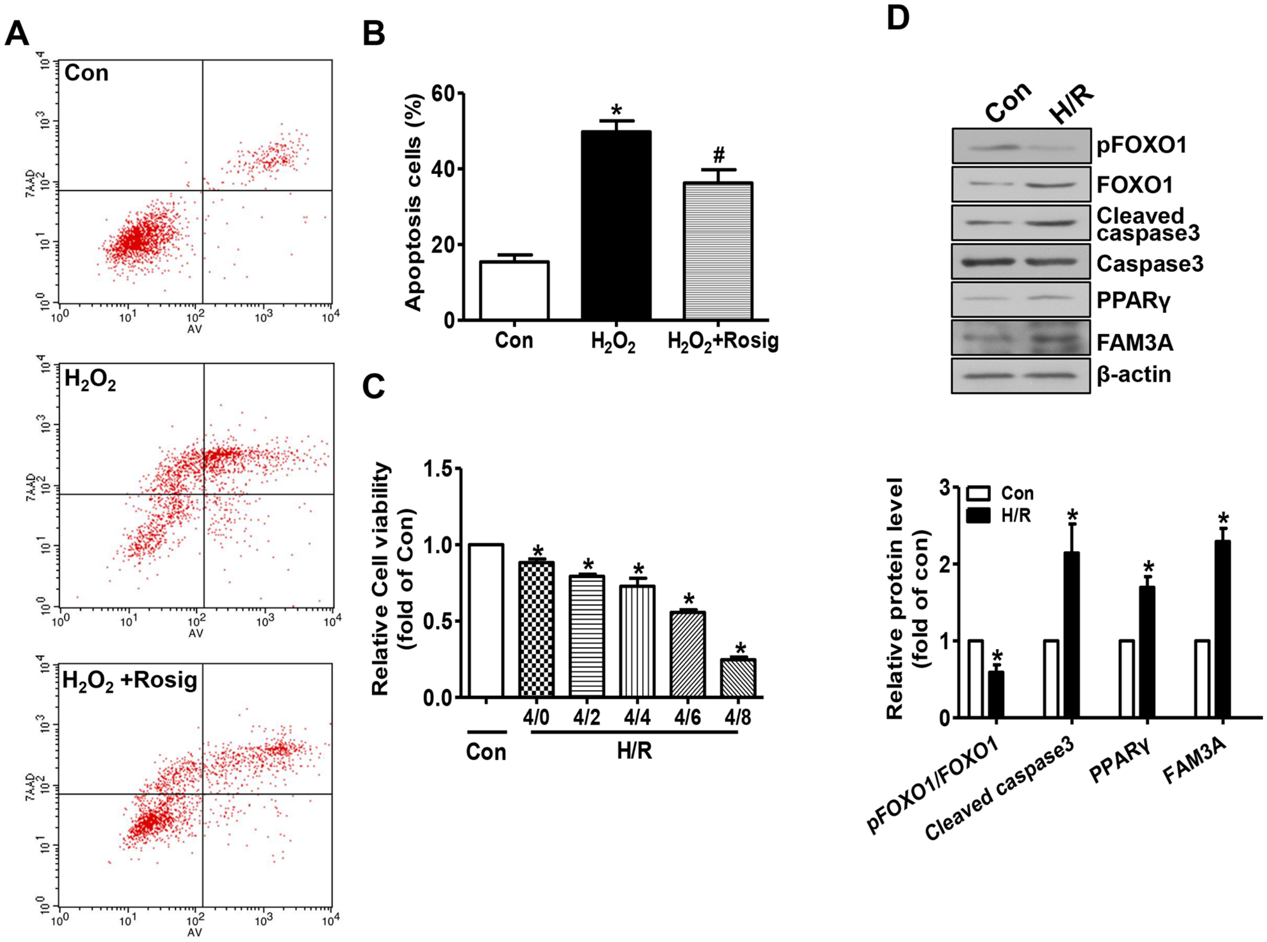

Supplementary Figure 13: Rosiglitazone treatment protected mouse hepatocytes against death induced by oxidative stress. (A-B) Rosiglitazone treatment ameliorated H_2O_2-induced mouse hepatocyte death. Representative flow cytometry data were shown in A, and quantitative data in B. N=3, *p<0.05 versus control cells, #<0.05 versus H_2O_2-treated cells. **(C)** Hypoxia/reoxygenation on cell viability of mouse hepatocytes. Mouse hepatocytes were exposed to hypoxia for 4 hours, followed by reoxygenation for various time length before cell viability were determined by MTT assay. H/R, hypoxia/reoxygenation. N=4, *p<0.05 versus control cells. **(D)** Hypoxia/reoxygenation activated PPARγ and FAM3A in mouse hepatocytes. Mouse hepatocytes were exposed to hypoxia for 4 hours, followed by reoxygenation for 6 hours before protein analyses. N=3, *p<0.05 versus control cells.

Supplementary Table 1: siRNA sequences against human and mouse FAM3A mRNA

Duplex name	Sense Seq	Antisense Seq
FAM3A-1(H)	5’-CAUGGAUCGUGGUCAGCAU-3’	5’-AUGCUGACCACGAUCCAUG-3’
FAM3A-2(H)	5’-AGAUCUGCCUCGAGGACAA-3’	5’-UUGUCCUCGAGGCAGAUCU-3’
FAM3A-3(H)	5’-GGCUGUAUCCCGCGGAGAA-3’	5’-UUCUCCGCGGGAUACAGCC-3’
FAM3A-4(H)	5’-UGGUGUUCGUGGCAUCCUA-3’	5’-UAGGAUGCCACGAACACCA-3’
FAM3A-1(M)	5’-UGAACUUCAAGAGAUCAUUGACAUC-3’	5’-GAUGUCAAUGAUCUCUUGAAGUUCA-3’
FAM3A-2(M)	5’-UAUUCGAAAGCUCAGGUGCUCUUCA-3’	5’-UGAAGAGCACCUGAGCUUUCGAAUA-3’
FAM3A-3(M)	5’-AUAAUGAUGAUUAGGGCCACGAUGC-3’	5’-GCAUCGUGGCCCUAAUCAUCAUUAU-3’

M:mouse H:human

Supplementary Table 2: List of oligonucleotide primer pairs used in real time RT-PCR and RT-PCR analysis

Target Gene	Sense Primer (5'-3')	Antisense Primer (5'-3')	Annealing temperature
FAM3A (M)	TCATGAGCAGCGTCAAAGAC	AGGGTACCTTCATGCAGTGG	59°C
PPARγ (M)	GACCAGCTGAACCCAGAGTC	GATGGCCACCTCTTTGCTCT	59°C
Bim (M)	GAGTTGGTTTCTATCCGTAAGC	GAACTAAGATTGTGAGGGTGGT	59°C
Bcl2 (M)	TCCTTCCAGCCTGAGAGCAA	TGACCCCACCGAACTCAAAG	59°C
Bax (M)	AGTGTCTCAGGCGAATTGGC	CACGGAAGAAGACCTCTCGG	59°C
FOXO1 (M)	ACATTTCGTCCTCGAACCAGCTCA	ATTTCAGACAGACTGGGCAGCGTA	59°C
P53 (M)	CCGACCTATCCTTACCATCATC	TTCTTCTGTACGGCGGTCT	59°C
Mdm2 (M)	GCAGAAGAAGGCTTGGATGT	TCGTCTTTGTCCTGCGTTTC	59°C
IP-10 (M)	AAGTGCTGCCGTCATTTTCT	GTGGCAATGATCTCAACACG	59°C
TNF-α (M)	CACAAGATGCTGGGACAGTGA	TCCTTGATGGTGGTGCATGA	59°C
MCP-1 (M)	AATGAGTAGCAGCAGGTGAGTG	GAAGCCAGCTCTCTCTTCCTC	59°C
IL-1α(M)	ATCAGCAACGTCAAGCAACG	GCTGATCTGGGTTGGATGGT	59°C
GAPDH (M)	aactttggcattgtggaagg	acattgggggtaggaaca	59°C

M: mouse; If not indicated, all the primer sequences are referred to mouse.
